# Usability and performance validation of an ultra-lightweight and versatile untethered robotic ankle exoskeleton

**DOI:** 10.1186/s12984-021-00954-9

**Published:** 2021-11-10

**Authors:** Greg Orekhov, Ying Fang, Chance F. Cuddeback, Zachary F. Lerner

**Affiliations:** 1 Department of Mechanical Engineering, Northern Arizona University, 15600 S McConnell Drive, NAU EGR Bldg 69, Flagstaff, AZ 86011 USA; 2 College of Medicine – Phoenix, University of Arizona, Phoenix, AZ USA

**Keywords:** Ankle, Exoskeleton, Incline walking, Stair ascent, Metabolic power, Cerebral palsy, Dorsiflexor assistance, Plantarflexor assistance

## Abstract

**Background:**

Ankle exoskeletons can improve walking mechanics and energetics, but few untethered devices have demonstrated improved performance and usability across a wide range of users and terrains. Our goal was to design and validate a lightweight untethered ankle exoskeleton that was effective across moderate-to-high intensity ambulation in children through adults with and without walking impairment.

**Methods:**

Following benchtop validation of custom hardware, we assessed the group-level improvements in walking economy while wearing the device in a diverse unimpaired cohort (n = 6, body mass = 42–92 kg). We also conducted a maximal exertion experiment on a stair stepping machine in a small cohort of individuals with cerebral palsy (CP, n = 5, age = 11–33 years, GMFCS I-III, body mass = 40–71 kg). Device usability metrics (device don and setup times and System Usability Score) were assessed in both cohorts.

**Results:**

There was a 9.9 ± 2.6% (p = 0.012, range = 0–18%) reduction in metabolic power during exoskeleton-assisted inclined walking compared to no device in the unimpaired cohort. The cohort with CP was able to ascend 38.4 ± 23.6% (p = 0.013, range = 3–132%) more floors compared to no device without increasing metabolic power (p = 0.49) or perceived exertion (p = 0.50). Users with CP had mean device don and setup times of 3.5 ± 0.7 min and 28 ± 6 s, respectively. Unimpaired users had a mean don time of 1.5 ± 0.2 min and setup time of 14 ± 1 s. The average exoskeleton score on the System Usability Scale was 81.8 ± 8.4 (“excellent”).

**Conclusions:**

Our battery-powered ankle exoskeleton was easy to use for our participants, with initial evidence supporting effectiveness across different terrains for unimpaired adults, and children and adults with CP.

*Trial registration* Prospectively registered at ClinicalTrials.gov (NCT04119063) on October 8, 2019.

**Supplementary Information:**

The online version contains supplementary material available at 10.1186/s12984-021-00954-9.

## Background

Ankle exoskeletons hold potential to augment walking performance in unimpaired individuals and in individuals with neurological conditions [1–4]. The ankle joint is a frequent target for powered assistance due to its critical role in efficient bipedal locomotion [5–7] and because it is a commonly affected joint in individuals with neurological deficits [8, 9]. Individuals with cerebral palsy (CP), for example, typically have ankle plantarflexor weakness and limited push-off power that contributes to slow, inefficient walking, particularly on graded terrain, like stairs [10–12].

Unburdened by the need to carry motors and a power supply, users walking with tethered ankle plantarflexor assistance have consistently demonstrated improved walking economy for nearly a decade [7, 13–15]. However, achieving improvements in walking economy with untethered ankle exoskeletons has apparently been more challenging, with only a small number of studies reporting activity performance benefits compared to walking without the device [1, 3, 16–18]. Untethered ankle exoskeletons capable of mobility augmentation outside of the laboratory follow two general design approaches: placing motors on the shank close to the joint or placing motors at the waist. Opting to minimize mass and the physical profile added to the lower-limb, Awad et al. [1, 17] developed a soft exosuit with waist-mounted motors that improved paretic limb function, walking speed and walking economy in stroke survivors. Mooney et al. [3] took a shank mounted motor approach instead, and addressed the metabolic detriment of adding mass distally on the leg by incorporating a clever mechanical design achieving high torque and power output, and demonstrated improvements in loaded and unloaded walking in healthy adults; this appears to be the only published work demonstrating a group-level improvement in energy efficiency in unimpaired individuals when walking with an untethered, battery-powered ankle exoskeleton compared to no device.

For several years, our group has worked on untethered, low-torque ankle exoskeletons for children and young adults with CP. We have demonstrated that bilateral assistance proportional to the user’s biological ankle moment during stance phase [19, 20] can improve key metrics such as energy expenditure and walking speed in small cohorts with CP during level walking [18, 21]. However, early prototypes had poor reliability and durability, and proved ineffective for individuals of body mass greater than approximately 45 kg because of limited torque production and significant motion of the ankle assembly relative to the shank and foot. Additionally, these prior exoskeletons were cumbersome to don and doff, designed without consideration for usability, and control was limited to a computer-based researcher interface. Usability factors are important yet under-researched aspects of wearable lower-limb exoskeleton design that hold practical implications for real-world deployment. Devices intended to augment mobility in the community should be easy to don and operate, with portable and intuitive user interfaces. The ability of individuals with CP to put on and operate an ankle exoskeleton without researcher or technician intervention remains unknown.

**Fig. 1:**
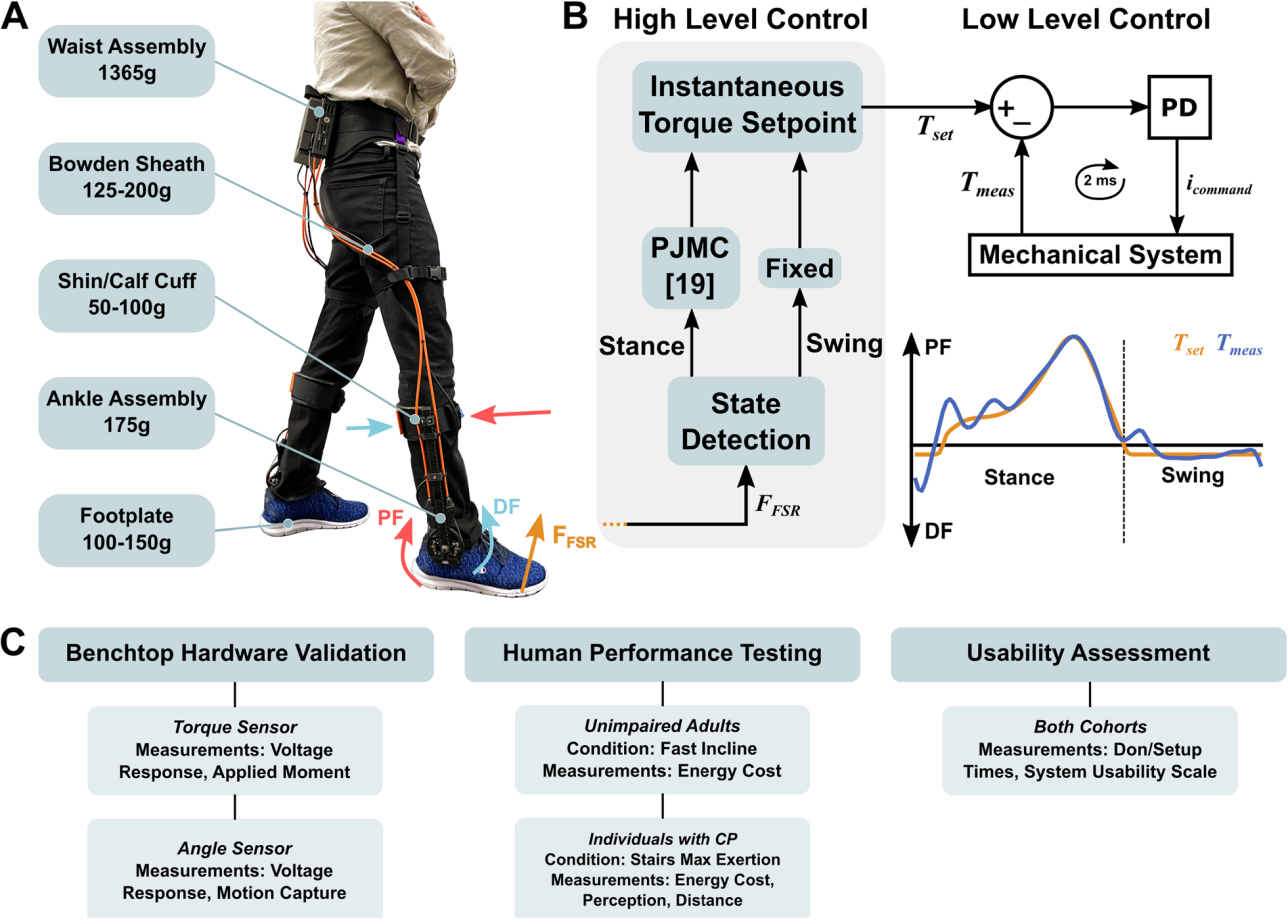
Exoskeleton mass breakdown, exoskeleton control overview, and protocol summary. **A** Device components and mass. Values are mass per leg except for the waist assembly. **B** High- and low-level control layers. The high-level controller was responsible for gait event detection (i.e., toe-off and heel strike) and assistive torque profile generation. During stance, a forefoot force sensor signal was an input to a proportional joint moment controller (PJMC) that generated an adaptive plantarflexor torque profile in real time [17]. During swing, a constant dorsiflexor torque was prescribed. The low-level controller tracked the plantarflexor and dorsiflexor torque profiles using a closed-loop PD controller. **C** A summary of the experiments, cohorts, conditions, and measurements analyzed in this study

The first goal of this study was to design a novel cable-driven ankle exoskeleton, validate custom torque and angle sensors, and evaluate electromechanical performance during ambulation (Fig. 1). Our second goal was to highlight the relevance and versatility of this device by demonstrating its ability to reduce the energy cost of fast incline walking in healthy adults, and on distance achieved during a maximal exertion stair-stepping exercise in CP. We selected these moderate- to high-intensity activities for these human performance experiments because we believe such activities reflect the real utility of ankle exoskeletons in both unimpaired and impaired populations, namely, augmenting ambulatory activities that have elevated ankle plantarflexor demand. We hypothesized both cohorts would have significant improvements while walking with vs without the device. Our final objective was to complete a usability assessment, quantifying the time for users or their caretakers, if applicable, to don and set up the device without researcher intervention. We hypothesized that individuals could don, calibrate, and receive assistance from the device in less than 5 minutes.

## Methods

### Exoskeleton design

We designed a lightweight bilateral, bidirectional battery-powered ankle exoskeleton (Fig. 1AB). Waist mounted motors actuated a pulley assembly at the ankle via a chain-to-cable transmission system. The instrumented ankle joint pulley was mounted within a carbon fiber tube that also supported cable housing reaction forces. Carbon fiber footplates and shank cuffs provided rigid yet comfortable load transfer interfaces. Sensors on the footplate informed a high-level controller used to provide adaptive plantarflexor torque during stance phase and/or constant dorsiflexor torque during swing phase (Fig. 1B). A custom embedded torque transducer at the ankle provided feedback for low-level closed-loop torque control. The total bilateral mass of the device ranged from 2.4 to 2.6 kg, depending on the cable length, and size of the footplates and cuffs (Table 1). The exoskeleton’s peak torque output was 30 Nm. Between 50 and 65% of the total exoskeleton mass (depending on the configuration) was contained within the waist assembly so that the detriment of distally added weight on metabolic power was minimized [22]. Mass minimization, modularity, comfort, and ease of donning and operation were important criteria that guided the design.

**Table 1:** Exoskeleton mass breakdown

Component^a^	Mass (kg)	Location on Body
Waist assembly	1.37	Waist
Cable transmission (×2)	0.31	Thigh
Ankle assembly and cuff (×2)	0.55	Shank
Footplate (×2)	0.28	Foot
Total Bilateral Exoskeleton Mass	2.51	

^a^The mass corresponding to components indicated with (×2) is bilateral. The exoskeleton mass breakdown presented was for a medium-sized exoskeleton sized for users between 160 and 185 cm tall

### Waist assembly and cable transmission

**Fig. 2:**
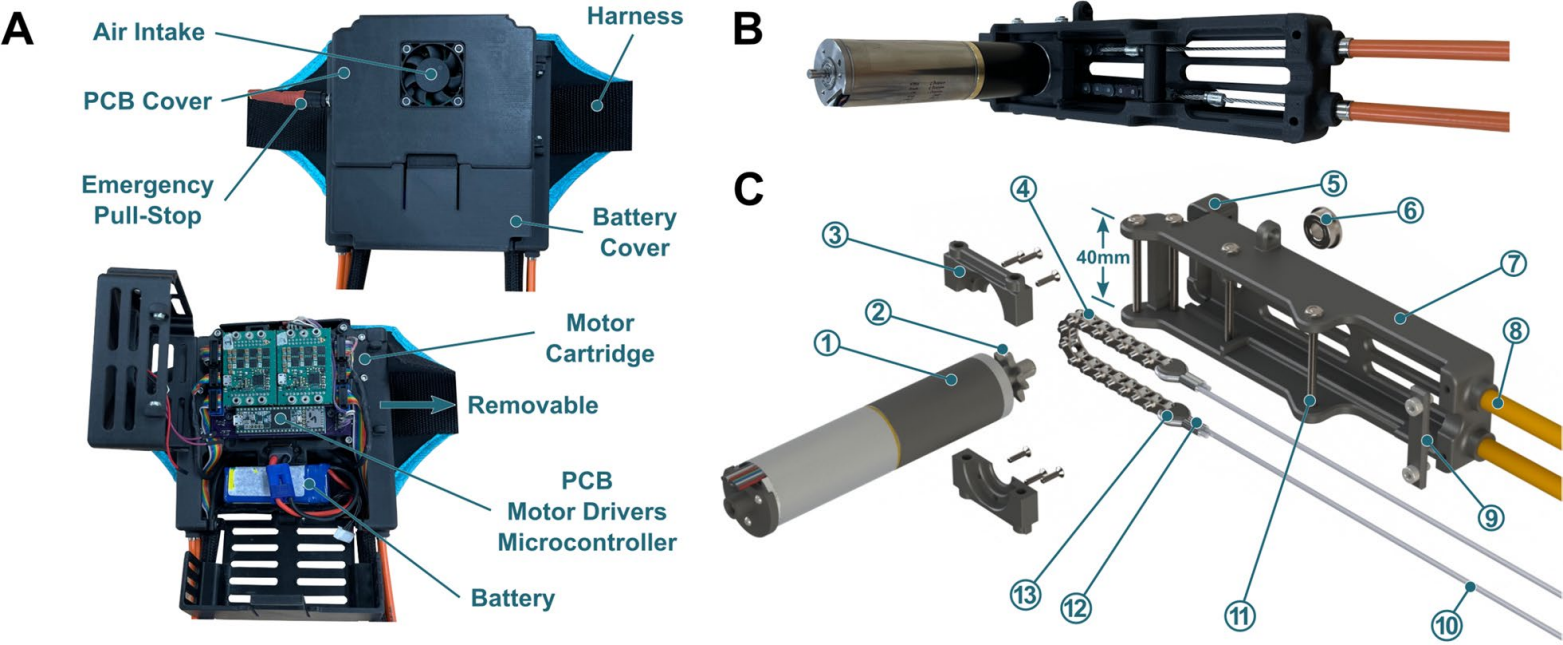
Waist assembly overview. **A** Closed and open pictures of the waist assembly module and harness system. The waist assembly module housed the motors, motor cartridges, PCB, battery, and wiring harness. **B** Assembled view of a motor cartridge assembly. **C** Exploded view of a motor cartridge assembly. (1) A 90 W Maxon motor with an 89:1 gearbox. (2) An 8-tooth sprocket welded onto the gearbox output shaft. (3) Reinforced motor blocks were the interface between the motor and cartridge. (4) A chain driven by the sprocket actuated the cable transmission. (5) A sliding cover on each cartridge permitted easy access to the chain assembly (6) A thrust bearing supported the motor shaft to prevent tip deflection during operation. (7) The cartridge was 3D-printed and reinforced with carbon fiber aligned with the long axis. (8) The Bowden sheaths guided steel cables down to the ankle assembly. (9) Wire strain relief. (10) Steel cable looped through final link on each side of the chain and passed through the Bowden sheath. (11) Steel bolts held the motor subassembly in place within the cartridge and attached the cartridge to the rest of the motor assembly. (12) A crimped swage held the steel cable looped through the chain. (13) A small guide component prevented cable stress concentrations and failure

The waist assembly housed the exoskeleton actuation and control hardware including the motors, custom printed circuit board (PCB), and battery (Fig. 2). A padded harness system fastened the assembly to the waist (Fig. 1A). A modular fiber-reinforced 3D-printed assembly casing was designed to mount two motors (EC4-Pole 90 W with 89:1 GP 22HP gearbox, Maxon) via cartridges and house the electronics module and battery (Fig. 2B). 18 mm sprockets were welded onto the gearbox output shafts and moved chains within the cartridge to actuate the cable transmission that rotated the ankle assembly, transmitting torque and power from the motor to the user. Steel cables were looped through the chain ends and were held in place by a guide and swage (Fig. 2C). Nylon webbing restraints held the cable transmission system to the thighs (Fig. 1A). Each motor mounting cartridge was removable, allowing for quick and easy replacement of each exoskeleton leg assembly independently. The cartridges were designed for ease of maintenance and to house different motor configurations and sizes. They could be quickly swapped for taller or shorter cable configurations depending on the user. Transmission cable configurations were made in set sizes to span a range of user heights (< 160 cm, 160–185 cm,  > 185 cm). The custom PCB interfaced with sensing, control, and wireless communication hardware including a microcontroller (Teensy 3.6, PJRC), motor drivers (ESCON Module 50/8, Maxon), Bluetooth module, and other components to regulate battery voltage and amplify measurement signals (e.g., INA125P, Texas Instruments). A 5 V cooling fan provided airflow through the motor assembly (Fig. 2A). A 24 V, 2000 mAh Li-Ion battery (KamPing) for this study was selected to provide an ambulatory duration equal to or greater than the typical physical therapy session (20–35 min [23, 24]) when walking near the peak torque rating.

### Ankle assembly

**Fig. 3:**
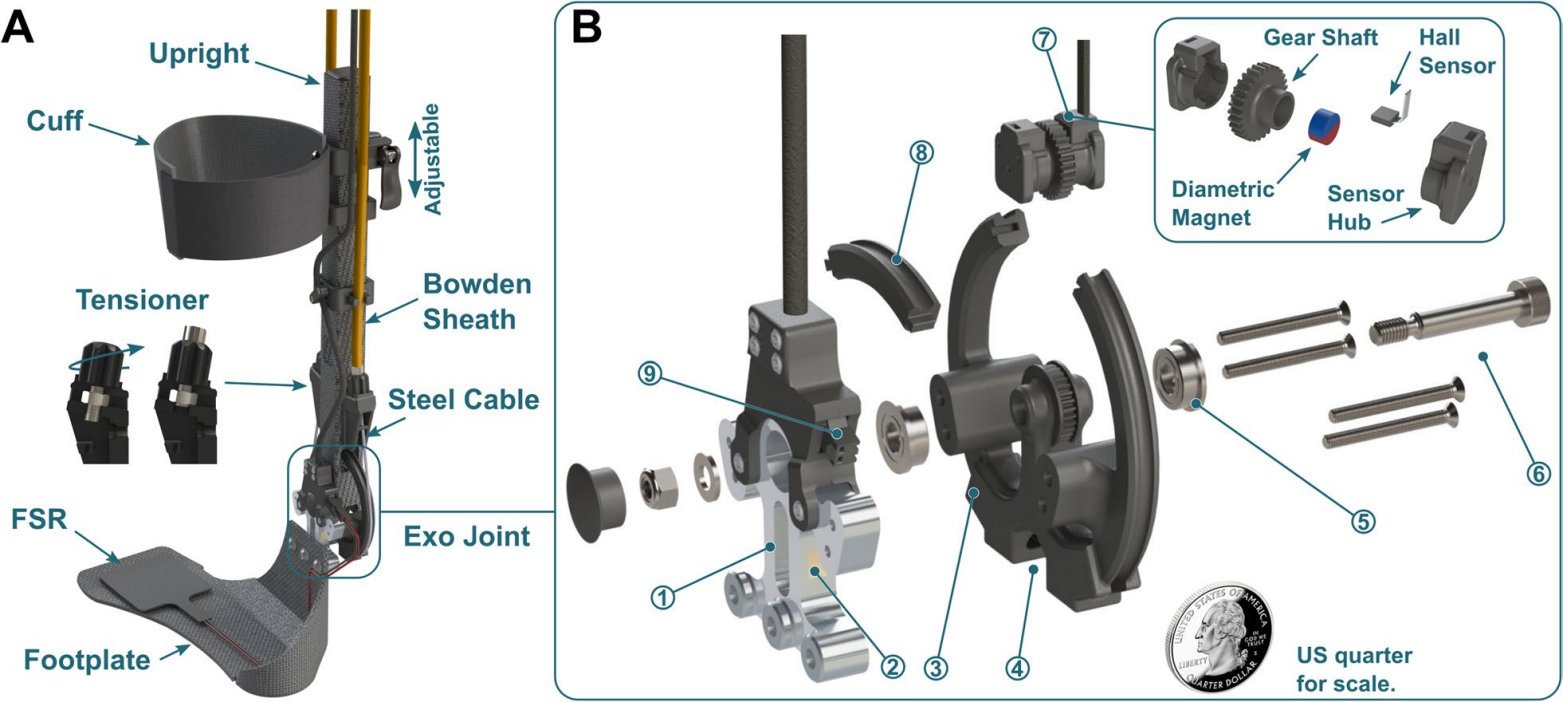
Ankle Assembly. **A** Assembled view of an entire ankle assembly, including calf cuff, machined carbon fiber upright, tensioners, instrumented exoskeleton joint, and footplate. Tensioners compressed the Bowden sheath via a pull-and-twist knob and kept the cable transmission taut. **B** Exploded view of the exoskeleton joint. (1) Torque transducer (2) Strain gage. (3) Carbon fiber-reinforced pulley. (4) Steel cable transmission crimping site. (5) Thrust ball bearings. (6) 6 mm shoulder bolt. Four steel bolts fixed the torque sensor to the pulley. (7). Angle sensor assembly and exploded view. A gear shaft meshed with the pulley rotated a diametric magnet underneath a stationary Hall sensor. The resulting voltage was used to calculate joint angle and angular velocity. (8) Removable pulley bridge allowing assembly within the carbon fiber tube. (9) FSR connection

The ankle assembly was designed to minimize distal mass and lateral protrusion from the shank, support cable transmission reaction forces, and provide a rigid interface to support and assist a user during activity. Mass added distally on the body increases the metabolic cost of walking more than when it is placed more proximally [22] and limits an exoskeleton’s theoretical potential for benefit [25]. Components placed on the medial portion of the lower limb increase the risk of inter-limb collisions, while posterior or lateral protrusions may cause collisions with the environment. Our previous prototypes suffered from a lack of assembly stiffness due to a large moment arm between the user and the lateral upright and the absence of out-of-plane stiffening geometry [16, 18, 26]. We addressed these issues through mechanical design and material selection specifically intended to maximize assembly stiffness, such as using a square carbon fiber tube for the upright, incorporating stiffening ridges to the footplate and cuff, and reducing lateral protrusion of the ankle assembly by designing a low-profile ankle joint with custom sensors (Fig. 3).

The ankle assembly incorporated a single degree of freedom rotational joint and interfaced with the user via a shank or calf cuff and a footplate (Fig. 3A). The steel cables rotated a torque- and angle-measuring pulley assembly (Fig. 3B). The pulley was placed within a carbon fiber tube and was supported on both ends by flanged bearings (Fig. 3B). The pulley was 80 mm in diameter and formed a 5:1 gear reduction with the motor sprocket. While the pulley design permitted 120 degrees of motion before colliding with the upright, the chain assembly limited the motion to 80 degrees which was sufficient to capture the biological ankle range of motion [27]. We designed a custom low-profile in-line torque transducer for low-level motor control and torque measurement (Fig. 3B) with the goal of minimizing the physical profile of the assembly and lateral lever arm. The lateral lever arm in this design was 3 cm measured from the center axis of the upright to the edge of the footplate vs. 5 cm for a previous prototype [16, 18, 28]. The lateral lever arm, and consequently both the coronal bending and axial twisting moments, were 40% smaller than on our previous devices. We also designed a custom embedded angle sensing unit that resided above the pulley within the carbon fiber tube to provide a platform for the development of new angle- or velocity-dependent control strategies, patient monitoring of ankle angle or range of motion, and measurement of the device’s mechanical power. The footplate was designed to be rigid but lightweight and had a curved feature to match the shape of the foot and metatarsals during toe-off [26]. Cuffs and footplates were made in set sizes, were easily swappable, and each footplate size spanned several shoe sizes [26]. A force-sensitive resistor (FSR, Flexiforce A502, Tekscan) placed on the footplate spanning the 1st through 3rd metatarsal heads under the ball of the foot was used by the microcontroller to detect gait events and generate real-time stance torque profiles.

The following subsections detail specific experiments related to the hardware validation, human performance testing, and usability assessment portions of the experimental protocol summarized in Fig. 1C.

### Torque sensor design

**Fig. 4:**
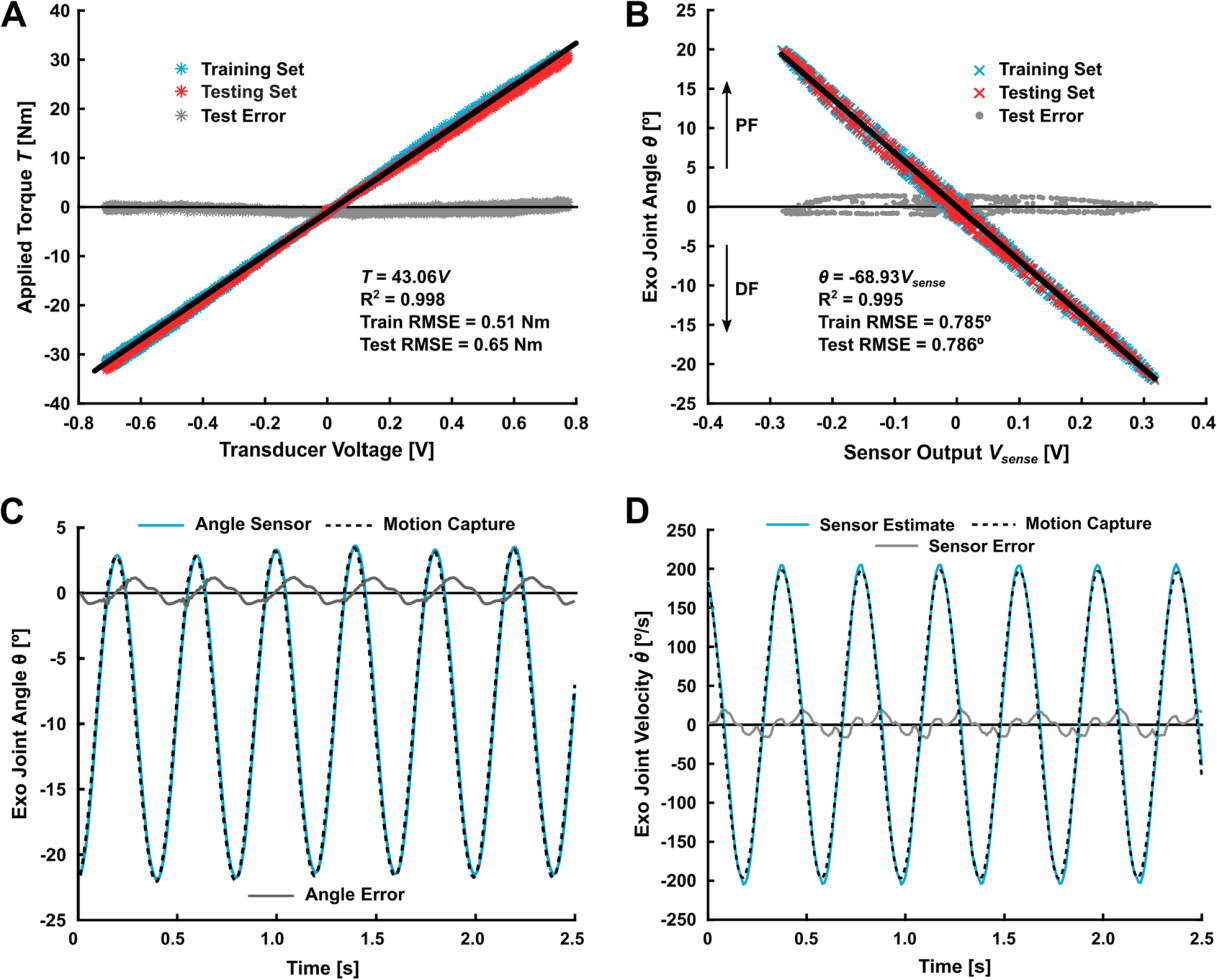
Torque (**A**) and angle sensor (**B**–**D**) validation results. **A** Linear regression for estimating torque applied to the transducer given a voltage measurement with root-mean-squared error (RMSE). Refer to Additional file 1 for torque sensor sensitivity to out-of-plane loads. **B** Linear regression relating angle sensor output to motion capture with RMSE; a positive angle corresponds to plantarflexion (PF). **C** Time series angle measurement with RMSE. **D** Comparison of sensor-estimated joint velocity to the motion capture result. Refer Additional file 1 for a comparison of sensor and motor velocity estimates during validation (Additional file 1: Fig. S5B)

Our custom torque transducer was a machined 7075-T651 aluminum part instrumented with strain gages designed to bi-directionally measure up to 30 Nm of torque (Figs. 3B,  4A). The thickness of the transducer was 10 mm and the mass was 30 g. For comparison, commonly used low-profile commercial sensors are over 25 mm thick and weigh over 50 g (e.g., Transducer Techniques TRT-500, [16, 18, 28–30]). The transducer measured the sagittal bending moment generated between the cable-driven pulley and footplate (Fig. 3, Additional file 1: Fig. S1). The full Wheatstone bridge strain gage configuration minimized the effects of temperature and out-of-plane loading [31], isolating sagittal-plane torque applied to the user’s ankle joint. The Wheatstone bridge voltages were measured, summed, and amplified using a differential op-amp and a 1-kOhm resistor (INA125P, Texas Instruments) on our custom PCB. Refer to the Torque Sensor Validation section in Additional file 1 for methods and figures related to the experimental setup for validating the torque sensor measurement and assessing its ability to isolate sagittal plane moments.

### Angle sensor design

An angle sensor was located above the pulley axis of rotation, residing within the carbon fiber tube to minimize the lateral protrusion of the assembly. The assembly consisted of a 3D-printed plastic gear shaft enclosed in hubs that meshed with a gear on the pulley (Fig. 3B). The gear shaft rotated a diametric magnet underneath a Hall Effect sensor (SS49E, Honeywell) in the lateral hub. A rotating diametric magnet in this configuration induced a repeatable sinusoidal voltage response from the Hall sensor [32]. The angle measurement was then used to estimate joint angular velocity in real time by numerical differentiation (Additional file 1: Fig. S4). Refer to the Angle Sensor Validation section of Additional file 1 for methods and figures related to the experimental setup for validating the sensor angle and velocity measurements.

### Software and control

The FSR signal was used to detect transitions between stance and swing, and determine an assistive torque profile during stance. A software threshold defined as a percentage of the total FSR signal range could be increased or decreased to adjust the initiation of stance phase and swing phase assistance. We used a high-level proportional joint moment controller (PJMC [19], Fig. 1) to generate an adaptive torque profile ($T_{set} \left( t \right))$ proportional to a real-time estimate of the biological ankle moment during stance phase, as in Eq. 1:

1 $$T_{set} \left( t \right) = T_{0} \frac{{F_{FSR} \left( t \right)}}{{F_{cal} }}$$

where $F_{FSR} \left( t \right)$ was the instantaneous FSR reading, $F_{cal}$ was the reference calibration value defined by the average of the peak FSR reading over three steps for each leg, and $T_{0}$ was the desired peak exoskeleton torque (e.g., 30 Nm). The footplate FSR captured the shape and magnitude of a signal that served as an estimate of the total biological ankle moment [19, 33]. $F_{cal}$ normalized the FSR signal, so any variance in the signal magnitude due to FSR placement on the footplate or foot contact with the FSR was eliminated and didn’t affect the real time torque profile $T_{set} \left( t \right)$. Nominal constant dorsiflexor assistance could be applied during swing phase. A low-level PD controller tracked the generated torque profile using measurements from the torque sensor at the ankle joint (Fig. 1B). PJMC was recently validated across variable terrain including inclined treadmill walking and stair ascent [33], and allows users to seamlessly transition between terrains. The same PJMC parameters were used for all participants and terrains in this study.

### Unimpaired cohort experiments

We recruited six healthy adults spanning a range of body sizes with the goal of demonstrating applicability of our device to unimpaired moderate-intensity walking performance augmentation (Table 2). During the experiment, participants walked for six minutes with and without exoskeleton assistance on a treadmill with a five-degree incline. We used a five-degree incline to mimic the maximum allowable ramp angle from Americans with Disabilities Act (ADA) guidelines [34]. We selected moderate intensity incline walking for our unimpaired performance testing experiment primarily because we believe it reflects the real utility of ankle exoskeleton in unimpaired populations, namely, augmenting moderate- to high-intensity ambulatory activities that have elevated ankle plantarflexor demand. Additionally, this condition satisfied our goal of demonstrating a potential benefit beyond the most commonly investigated terrain (level ground). Participants used the shortest exoskeleton configuration that allowed them to walk without limiting step length. Participants were also sized for footplates and cuffs that fit snugly and were comfortable. Proper footplate fit was qualified by contact between the ball of the foot and the footplate FSR and by close alignment (within 3 cm) of exoskeleton and biological ankle joint centers. Footplate mounting hole patterns allowed for easy joint center alignment. Extra foam padding was added to footplates and cuffs as needed for comfort and fit.

**Table 2:** Unimpaired participant information

Participant	Sex	Age (years)	Mass (kg)	Height (cm)	Stance torque (Nm)	Swing torque (Nm)	Walking speed (m/s)	Trial order
P1^a^	F	24	50.0	160.0	17.5	2.5	1.25	Exo-Shod
P2	F	22	57.5	152.5	20.5	3.0	1.25	Shod-Exo
P3	M	26	91.6	173.0	30.0	3.0	1.25	Shod-Exo
P4	M	22	65.0	162.6	23.0	3.0	1.25	Exo-Shod
P5	F	23	45.7	155.0	16.0	2.5	1.35	Exo-Shod
P6	M	20	72.6	178.0	25.0	2.5	1.15	Shod-Exo

^a^This participant also completed a short exoskeleton-assisted walk on a step mill. Her typical exoskeleton torque, angular velocity, and power for inclined walking and stair ascent are shown in Fig. 5. Additional participant torque, velocity, and power curves are available in Additional file 2

**Fig. 5:**
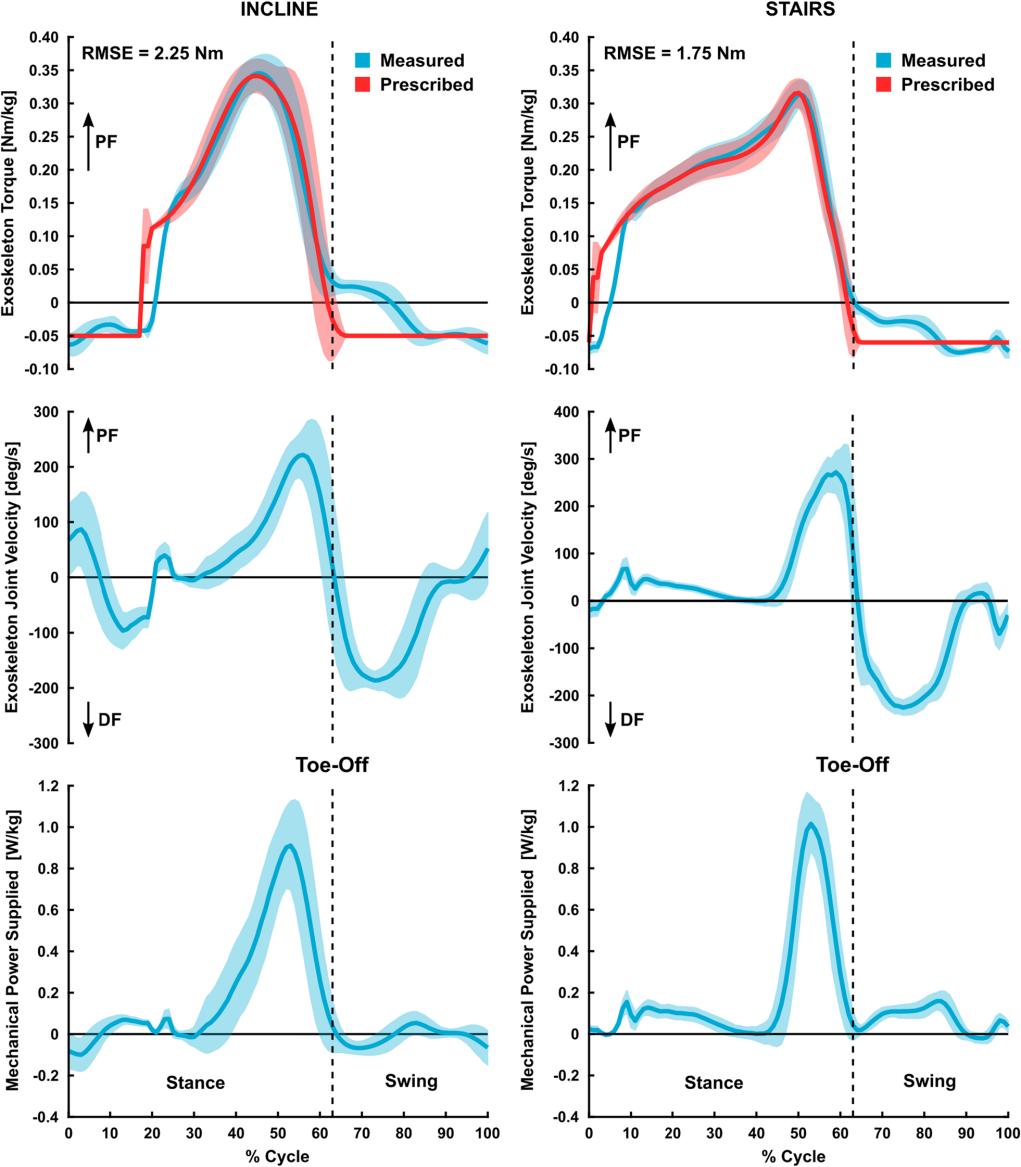
Representative exoskeleton measured (blue) and prescribed (red) torque (top row), velocity (middle row), and power (bottom row) profiles (mean ± standard deviation) from a single subject (P1, Table 2) during assisted walking on a 5-degree incline (left column) and on a stair-stepping machine (right column). Mechanical power (bottom row) was calculated by multiplying measured exoskeleton torque (Nm) and angular velocity (radians per second). Torque and power were normalized by the participant’s body mass. Torque tracking error during early stance and immediately after toe-off are due to motor torque rate and speed limitations. Refer to Additional file 2 for additional participant torque, velocity, and power curves and to Additional file 3 for a short section on power consumption and electrical to mechanical power efficiency

Prior to the first trial, participants were given 10–15 min of exoskeleton acclimation time during which an operator tuned the exoskeleton assistance and treadmill speed. After a standing torque sensor zero calibration, participants walked at 1.0 m/s with 0.35 Nm/kg of nominal peak stance phase assistance and 0.05 Nm/kg of swing phase assistance while the dynamic FSR calibration was performed. Torque levels were chosen from our prior works [18, 21, 28] and pilot tests. Then, the operator increased the treadmill speed until the participant confirmed that the activity was of moderate intensity (Table 2). The operator re-calibrated $F_{cal}$ to ensure good torque tracking at the faster walking speed, modified the FSR state transition threshold to ensure timely transitions between stance and swing (if needed), and adjusted swing phase assistance until the participant confirmed that the dorsiflexor torque was helpful after toe-off but did not impede the following heel strike. Most participants were comfortable with exoskeleton assistance after about 5–10 min of acclimation time. The calibrated exoskeleton parameters were saved and used during the shod-exoskeleton comparison experiment.

Each participant was assigned one shod and one assisted trial; trial order was alternated across the cohort (Table 2). We collected metabolic data using an indirect calorimetry unit (K5, COSMED). Oxygen and carbon dioxide volumes were used to calculate metabolic power using Brockway’s equation [35] for the last 3 min of each trial [36, 37]. Prior to each trial, the participant stood quietly for 2–3 min or until respiratory data were steady. The last minute of respiratory data during standing prior to each trial was used to calculate basal metabolic rate. The metabolic power for each walking trial was offset by the basal rate and normalized by body mass to calculate net metabolic power [38]. Between trials, participants sat and rested for 10 min.

We streamed exoskeleton signals, including motor current and velocity, desired and actual joint torques, and exoskeleton joint angle and angular velocity, to a custom MATLAB (R2018b, MathWorks) interface at 100 Hz. Exoskeleton joint power was calculated as the product of the measured joint torque and angular velocity for each leg. The net metabolic powers between the shod and exoskeleton trials were compared to assess the impact of powered exoskeleton assistance on energetics.

### Impaired cohort experiments

We recruited seven individuals with CP spanning a range of ages and impairment levels with the goal of demonstrating that our device can be effective at improving aerobic capacity during a maximal exertion test in this patient population (Table 3). Moving beyond our prior research that focused on augmenting walking on level ground in CP, we sought to explore application of ankle exoskeleton assistance to improve maximum exertion performance, which has not been previously explored in the literature. We designed an experiment to test performance on a stair-climbing machine as a way to expanding our understanding on the use of ankle assistance across different terrains and ambulatory intensities. Individuals with CP have difficulty with stair ascent [39] and are acutely susceptible to lower leg muscle fatigue [40], so we sought to demonstrate that our device was effective at prolonging the duration of this high-intensity activity. Inclusion criteria for this experiment included diagnoses of CP; the ability to walk on a stair machine for at least 5 min; Gross Motor Function Classification System (GMFCS) level I, II, or III; at least 20° of passive ankle plantarflexion range of motion; no knee extension or ankle dorsiflexion contractures greater than 15°; no orthopedic surgery completed in the prior 6-month period; and the absence of any medical condition other than CP that would affect safe participation. Participants were fitted with an ankle exoskeleton, footplates, and cuffs as described in the previous section. The same exoskeleton calibration procedure as in the previous section was conducted prior to exoskeleton-assisted trials.

**Table 3:** Impaired participant information

Participant	Sex	Age (years)	Mass (kg)	Height (cm)	GMFCS^a^ level
CP1	M	33	71.4	170	II
CP2	M	11	48.4	150	I
CP3	M	15	57.2	165	I
CP4	F	25	47.4	147	III
CP5^b^	M	14	39.5	148	II
CP6^b^	M	12	37.7	141	II
CP7	M	14	55.8	165	II

^a^GMFCS, Gross Motor Function Classification System. ^b^Two participants did not perform the maximal exertion portion of the experiment because of minimum mass requirements on the stair-stepping machine

The maximal exertion test protocol was as follows. The stair stepping rate was increased from each participant’s comfortable rate by one intensity level (0.3–0.4 floors/min) every thirty seconds until the participant indicated they wanted to stop. All participants wore a safety harness and were surrounded by researchers ready to stop the machine and support the participant to prevent harm. Participants completed one shod and one exoskeleton-assisted maximal exertion trial (Additional file 4: Video S1). Participants took a 20-min break between trials and confirmed that they were fully rested. Two of the seven participants were too light to trigger an increase in stair stepping rate and were unable to complete the experiment. We prescribed 0.30 Nm/kg of nominal peak plantarflexor assistance and 0.03 Nm/kg dorsiflexor assistance. During each trial, we recorded duration, step rate, and metabolic rate. We calculated the total distance travelled in number of floors (1 floor = 16 steps). After each trial, we recorded each participant’s perceived exertion using standard scales [41]. We compared the floors ascended between the shod and exoskeleton conditions. We also compared net metabolic power between the conditions over the duration of the shortest trial because intensity increased as the trials continued and we sought to make a direct comparison of metabolic power for the same duration and intensity. For example, if the shod trial was 5 min long and the assisted trial was 7 min long, we compared the average net metabolic power across the first 5 min of both trials.

### Usability assessment

All seven participants with CP performed a device usability assessment with the goal of demonstrating that time to don and operate the device improved with practice and could be completed in less than 5 min. Our usability experiments were motivated by our interest in conducting future evaluations of ankle exoskeleton assistance for augmenting mobility in free-living scenarios. The cohort included a wide range of participants as we were interested in receiving a variety of feedback from both children and adults on the usability and effectiveness of our exoskeleton. We recorded the time of each step of the donning process and the total app setup time (from powering on the device to walking with full torque magnitude) for each participant, including time spent reading instructions. Participants completed the exercise three times. Three individuals from the unimpaired cohort (P1, P3, and P4) were selected to perform the same assessment as a reference. Donning our device was a three-step process. Participants (1) prepared the device by placing it on a chair and placing footplates within shoes, (2) inserted their feet into the shoes, tied the shoes tightly, and strapped the cuffs in place on each shank, and (3) clipped and adjusted the waist assembly straps (Additional file 4: Video S2). We designed a custom iOS application that would automatically connect to the exoskeleton and guide the user through steps needed to start walking with assistance. Controlling the device using the iOS app was a quick three-step process that included: (1) user weight input in pounds, (2) static torque transducer calibration during quiet standing, and (3) dynamic controller calibration (state detection and ankle moment normalization) while walking in zero-torque mode. When ready, participants were instructed to walk and the firmware automatically completed the walking calibration and provided 0.30 Nm/kg of nominal peak stance torque and 0.03 Nm/kg swing torque, building in magnitude from zero over the course of three steps per leg. Refer to Additional file 4 for a link to the donning instructions on our website. The most affected participant (CP4) received parental assistance due to severe upper-extremity disability. To assess subjective user experience and quantify the usability of the exoskeleton, each participant completed the System Usability Scale questionnaire [42]. Briefly, the System Usability Scale includes 10 statements rated by means of a 5-point Likert scale, from 1 (strongly disagree) to 5 (strongly agree), and the scores have a range of 0 to 100 that is divided into five scales: score of 0–25: worst, score of 25–39: poor, score of 39–52: OK, score of 52–85: excellent, and score of 85–100: best imaginable [43].

### Statistics

All data sets were tested for normality using Shapiro–Wilk tests at the 5% significance level [44] and all samples were normally distributed. We compared net metabolic power for the unimpaired cohort and net metabolic cost of transport and distance travelled for the impaired cohort between shod and exo-assisted trials. For the usability section, we compared time needed to don and setup the device across three attempts. Two-tailed paired t-tests were used to assess differences at 5% significance for all group-level comparisons. Cohen’s d (d) was used to calculate effect size as the difference of group means divided by the pooled standard deviation, where 0.2 was considered a small effect, 0.5 a medium effect, and 0.8 a large effect [45]. All analyses and statistical comparisons were done in MATLAB. Simple statistical comparisons were used without p-value corrections so that the reader may judge the significance and impact of the group-level comparisons for themselves.

## Results

### Torque sensor validation

The linear model relating our custom torque sensor’s voltage output to applied torque explained 99% of the data variance and had low overall mean testing error and variance between predicted and actual torques (Fig. 4A). The torque absolute test root-mean-squared error (RMSE) was 0.65 Nm. We assessed the ability of the torque sensor to isolate sagittal plane moments and confirmed that out-of-plane sensitivity was between 5.2% and 15.5% of the sagittal bending moment sensitivity (Additional file 1: Fig. S3). No evidence of fatigue or offset drift has been observed.

### Angle sensor validation

The linear model relating our custom angle sensor’s voltage output to the motion capture angle explained 99% of the data variance and had low overall mean testing error and variance between predicted and actual pulley angles (Fig. 4B). The angle and angular velocity absolute RMSE computed from the ankle joint sensor relative to motion capture was 0.67 degrees and 9.01 deg/s, respectively, for a 2.5-s sample (Fig. 4CD). Estimating the exoskeleton joint velocity using measured motor velocity and the transmission system gear reductions yielded large error compared to motion capture (RMSE = 17.85 deg/s, Additional file 1: Fig. S4B).

### Unimpaired cohort experiments

**Fig. 6:**
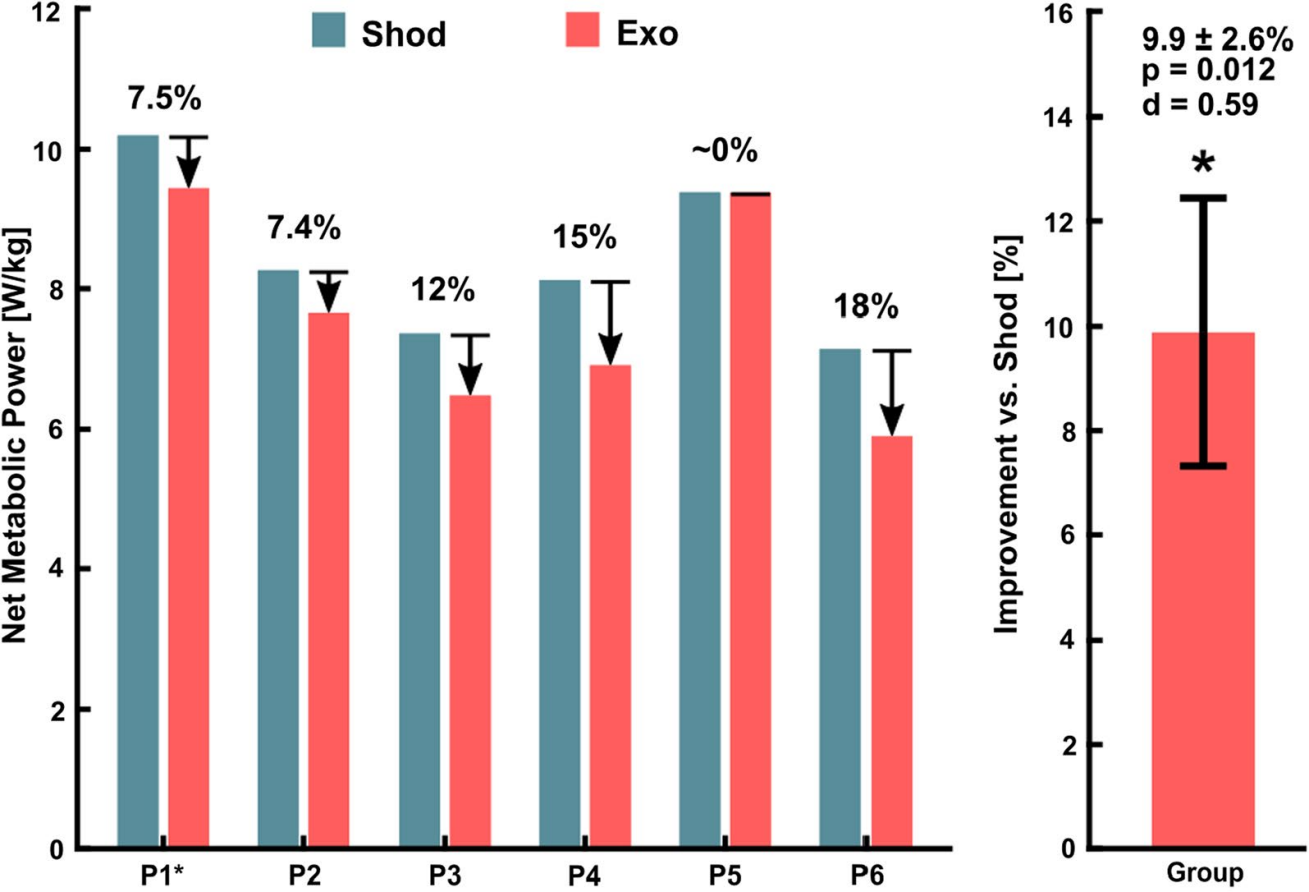
Unimpaired cohort metabolic results during incline treadmill walking with and without the exoskeleton. Five of the six participants responded well to exoskeleton assistance and showed a reduction in net metabolic power compared to no device. Refer to Table 2 for participant information and trial order. *Significant at 95% confidence

Five of the six unimpaired participants responded well to exoskeleton assistance during inclined treadmill walking (Fig. 6). Improvements in energy cost during the last 3 min of the trial ranged from 7.4% to 18%, with one participant (P5) showing no change when walking with assistance. Our cohort had a 9.9 ± 2.6% (mean ± standard error) improvement in metabolic power when walking with vs without the device (p = 0.012, d = 0.59, Fig. 6). Exoskeleton torque, angular velocity, and mechanical power were captured for five of the six unimpaired participants (Fig. 5, Additional file 2).

### Impaired cohort experiments

**Fig. 7:**
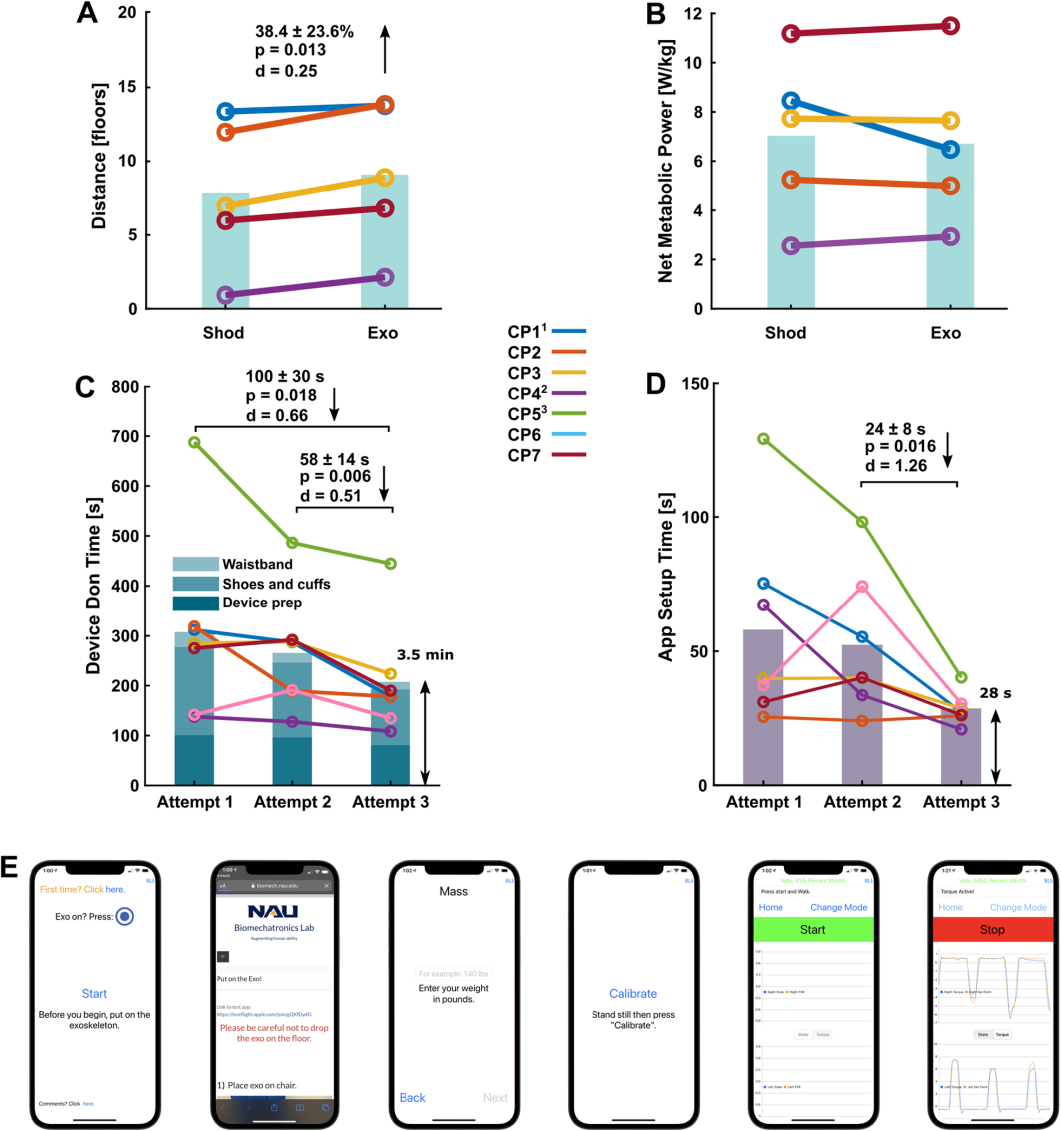
Maximal exertion stair-climbing results (top row) and usability assessment (bottom row). **A** Number of floors climbed during the maximal exertion experiment. **B** Average net metabolic power during the max exertion experiment. In general, participants had little change in energy cost but climbed more floors when walking with the device. ^1^CP1 had a large reduction in net metabolic power without a change in floors travelled suggesting that he likely could have climbed higher. **C** Device don time. The final attempt time was significantly lower than both the first and second attempts. ^2^A caregiver assisted CP4 throughout the assessment. ^3^CP5 had spastic hemiplegia of the upper and lower-extremities and had difficulty handling the device, but had a nearly three-minute improvement with practice. **D** App setup time. Setup time decreased with practice to ~ 30 s. **E** App at each step. From left to right: (1) start screen checks to see if a device is in range, (2) optional donning instructions (link available in Additional file 4), (3) mass input, (4) torque sensor calibration, (5) screen for adjusting parameters and starting trial, (6) example of an active trial

All participants were able to safely complete the maximal exertion stair-climbing test without incident. The number of floors climbed during the maximal exertion experiment increased by 38.4 ± 23.6% with exoskeleton assistance compared to shod (p = 0.013, d = 0.25, Table 4, Fig. 7A). Despite the increase in distance ambulated, the net metabolic powers for assisted and unassisted maximal exertion tests were not significantly different (p = 0.49, Fig. 7B), and perceived exertion on a scale of 1–10 was similar (6.8 ± 0.8 with the device vs 7.2 ± 1.5 without the device, p = 0.50).

**Table 4:** Maximum exertion speed and distance results

Participant	Stair speed (floors/min)^a^			Floors Ascended		Improvement over Shod (%)
	Start	Shod End	Exo End	Shod	Exo	
CP1	2.7	5.2	5.2	13.37	13.77	3
CP2	3	4.1	4.6	11.97	13.85	15.7
CP3	2.7	4.1	4.6	6.95	8.89	27.9
CP4	1.2	1.6	2	0.92	2.13	131.5
CP7	2.7	3.7	3.7	5.97	6.81	14.1

^a^Increasing exercise intensity on the step mill increased the ascent speed incrementally

### Usability assessment

The usability assessment results showed that total don time and app setup time generally decreased with practice (Fig. 7DE). Placing the footplates into the shoe and donning the ankle assembly was the most time- consuming step. The third attempt time (3.5 ± 0.7 min) was significantly different from the first attempt (down 100 ± 30 s, p = 0.018, d = 0.66) and from the second attempt (down 58 ± 14 s, p = 0.006, d = 0.51). Refer to Additional file 4: Video S2 for an example of the usability assessment. For reference, the average unimpaired final don time was 1.5 ± 0.2 min. The final app setup time (28 ± 6 s) was significantly different from the second attempt (down 24 ± 8 s, p = 0.016, d = 1.26). For reference, the average unimpaired final app setup time was 14 ± 1 s. The average System Usability Scale questionnaire score of the impaired cohort was 81.8 ± 8.4 (“excellent”).

## Discussion

The goal of this study was to design a highly useable lightweight ankle exoskeleton with custom sensing and provide an initial indication of its effectiveness across a range of terrains and users. Our design utilized a modular motor assembly mounted at the waist to provide ankle torque via interchangeable Bowden cables, calf cuffs, and footplates in standard sizes that spanned a wide range of users from children to adults. We validated our in-line sensing modules that provided direct, real-time measurement of ankle angle, torque and power; these sensors provide an opportunity for the development of new control strategies (e.g., velocity-dependent muscle force prediction), patient monitoring (e.g., ankle range of motion), closed-loop torque control, and assessment of mechanical performance (e.g., torque tracking). The device was able to provide up to 30 Nm of peak torque with a mass 2.3–2.6 kg depending on size and battery selection. We developed a custom iOS application allowing users to control the device themselves. We accept our primary hypotheses that (1) improvements in ambulatory performance (economy or distance) for both unimpaired and impaired cohorts are detectable during diverse exoskeleton-assisted tasks, and (2) users can don and control the device in less than 5 min. While the improvements in walking economy and distance are motivating, we caution the reader to interpret the results with care due to the small cohort sizes.

Our battery-powered device improved moderate-intensity incline walking efficiency by 10% compared to without wearing the device in an unimpaired cohort of adults of diverse body masses and statures (Fig. 6). Compared to untethered and even tethered systems, our device had competitive peak torque capability when normalized to the mass added onto the shank (Additional file 2: Fig. S2) and demonstrated similar improvements in energy expenditure. For example, the seminal untethered, shank-mounted exoskeleton from Mooney et al., weighed 1.06 kg per leg, produced up to 45 Nm (0.5 Nm/kg) peak torque during unloaded, level walking for a small cohort of heavy unimpaired adults (n = 6, mass = 89 ± 8 kg, mean ± SD) and induced a group-level reduction in energy expenditure of 11 ± 4% (mean ± SE) compared to walking without wearing the device [3]. On the other side of the spectrum, an untethered soft exosuit from Awad et al. with a peak torque of about 15 Nm (0.15 Nm/kg) and a distal mass of 0.42–0.50 kg was successful in preliminary and clinical trials during unilateral assistance with stroke survivors [1, 17]; we are not aware of bilateral unimpaired metabolic results for this system. A tethered ankle exoskeleton from Galle et al. had a peak torque of 36.6 Nm (0.6 Nm/kg), a distal mass of 0.445 kg per leg, and induced a group-level reduction of 12.3 ± 2.9% compared to shod (n = 14, mass = 61.0 ± 4.5 kg) [14]. A high-powered tethered exoskeleton from Zhang et al. with distal mass 0.875 kg induced a 21% improvement in a single subject during walking at a 10-degree incline [15] whereas, in the present study, the maximum observed reduction in energy cost during 5-degree incline walking was 18%. Compared to the aforementioned exoskeletons, our device produced up to 30 Nm, had a distal mass of 0.415 kg per leg, and induced a similar group-level reduction in energy consumption in an unimpaired cohort with greater mass variability than the aforementioned studies (mass range of 46–96 kg). As far as we are aware, our device had one of the lightest ankle assemblies of any research or commercial powered device at the time of this writing. The light distal mass likely contributed significantly to the observed benefits in energy reduction based on the augmentation factor, an estimate of potential benefit that balances positive power production with detriments to energy consumption due to added mass [25, 46]. Though our exoskeleton provided less torque and power than the device described in Mooney et al. [3], it had a similar augmentation factor due to its reduced distal mass (between 28 and 56 W vs. 44 W in [46] and 33 W in [25]). Augmentation factor was calculated as described in Mooney et al. [25] using our in-line joint sensors. Refer to Additional file 5 for an example calculation of augmentation factor.

Maximal exertion tests are commonly used to assess functional capacity, with individuals with CP having lower maximal oxygen consumption compared to unimpaired individuals [47]. In this study, we demonstrated that children and young adults with CP were able to ascend almost 40% more steps on a stair stepping machine while using the device during a maximal exertion test. This provides new insight into the potential for wearable ankle assistance to provide both psychological and physiological benefits during high-intensity activities (Fig. 7CD). For example, average metabolic power or perceived exertion remained the same despite the encouraging group-level increase in distance. One of the most promising findings was that our most impaired participant (CP4) ascended nearly twice as far with exoskeleton assistance compared to no device (2.1 vs. 0.9 floors, respectively). An interesting result at the individual level was that while CP1 had only a very small improvement in distance when using the device, there was a sizeable reduction in metabolic cost compared to no device (~ 23%, Fig. 7B), suggesting that he opted to decrease his effort while using the device as opposed to maintaining the same level of effort when not using the device like most participants. While additional research is needed, our results suggest that lightweight untethered ankle assistance may allow for increased and task-oriented training in individuals with CP, which has been proven successful at improving mobility in both CP and post-stroke patients [48–50].

To the best of our knowledge, this is the first study to report on the design of an untethered exoskeleton that allows individuals with physical disabilities to don and operate the device without researcher intervention. Individuals with CP were able to don and control the device to the point of walking with assistance (either on their own or with caregiver aid) in an average of just 4 min, suggesting that future deployment in clinical and home settings may be realistic. While the mobile app allows the user to modify exoskeleton tuning parameters, we envision that the user would not need to adjust tuning parameters after the initial device fitting and tuning process with a trained physical therapist or researcher. The rate of improvement over three attempts suggests that donning and setup time would likely decrease further with continued use. For example, one participant (CP5), a determined teen with upper- and lower-extremity spastic hemiplegia, had considerable difficulty donning the device on his own but improved his time by almost 3 min with practice (Fig. 7D). When prompted for feedback on the design, comfort, and control of the exoskeleton, users and caregivers tended to comment favorably on the low hardware profile on the shank and waist, low device weight, shank and waist interface comfort, quick don and doff times, and intuitive torque assistance. Donning the shoes and cuffs was similar to typical AFOs and the waist straps only needed to be adjusted once. The System Usability Scale questionnaire score of 81.8 ± 8.4 (excellent) was comparable to a commercial robotic device (Ekso GT, score: 83) and better than another pre-market devices (PASfinal, score: 59.5) [51].

While small cohort exoskeleton studies are common [3, 13, 14, 25, 52], we acknowledge that a limitation to this study was the small sample sizes used for statistical analysis and encourage the reader to interpret group-level results and statistical significance with caution. Another limitation of this study was that the battery used during the experiments provided just 37 min of walking duration at the average torque used across both cohorts (~ 22 Nm). While we met and exceeded our goal of completing performance testing with a battery meeting the typical ambulatory duration of a standard physical therapy session (20–35 min [23, 24]), the battery used in this study would be insufficient for all day use. However, we theorize that in the future deployment of this or similar commercial exoskeleton systems, users may prefer to minimize adding mass to the body by interchanging multiple batteries throughout the day.

## Conclusion

This study reported on the design and initial testing of a new, lightweight ankle exoskeleton. We validated custom low-profile joint-level sensing and achieved one of (if not) the lightest reported distal mass placements of any tethered or untethered powered ankle exoskeleton. The device performed well across a range of ambulatory conditions, walking abilities, and body sizes, reducing incline walking energy consumption in unimpaired adults and improving maximal exertion stair climbing distance in children and adults with CP. Our device was effective for children with CP as light as 38 kg and unimpaired adults as heavy as 92 kg, suggesting that 0.30–0.35 Nm/kg peak plantarflexor assistance and 0.03–0.05 Nm/kg dorsiflexor assistance was sufficient to improve ambulatory performance. All of our unimpaired participants and most of our participants with CP were able to don and initiate user-calibrated ankle assistance in under 5 min without researcher intervention. Future work will continue to explore the effectiveness of adaptive ankle exoskeleton assistance across a multitude of challenging terrains for unimpaired and impaired participants.

## Supplementary Information

**Additional file 1:** Supplemental figures detailing hardware validation methods and results.

**Additional file 2:** Supplemental exoskeleton performance data. P4's torque, velocity, and power curves for inclined walking and stair ascent. Comparison of our device to other prominent exoskeleton studies.

**Additional file 3:** Exoskeleton power consumption and electrcal-to-mechanical power efficiency analysis.

**Additional file 4:** Video and instruction links. Links to videos of exoskeleton maximal exertion and usability experiments. Link to exoskeleton donning instructions.

**Additional file 5:** Augmentation factor calculation for unimpaired cohort.

## Data Availability

The datasets used and analyzed during the current study are available from the corresponding author on reasonable request.

## Abbreviations

CP: Cerebral palsy
PCB: Printed circuit board
GMFCS: Gross Motor Function Classification System
RMSE: Root-mean-squared error
PF: Plantarflexion (refers to angle)
DF: Dorsiflexion (refers to angle)
