# Supplementary material for: Usability and performance validation of an ultra-lightweight and versatile untethered robotic ankle exoskeleton
Source: J Neuroeng Rehabil. 2021 Nov 10;18:163. doi: 10.1186/s12984-021-00954-9 (PMC8579560; doi:10.1186/s12984-021-00954-9)
Supplement: Supplementary file 1 — Additional file 1: Supplemental figures detailing hardware validation methods and results. [file 12984_2021_954_MOESM1_ESM.docx]

Torque Sensor Validation

The torque sensor was instrumented with a full Wheatstone bridge, which is a gage configuration that negates out-of-plane loads and temperature effects. The transducer measured the sagittal bending moment generated between the cable-driven pulley and footplate (Fig. S1).

| 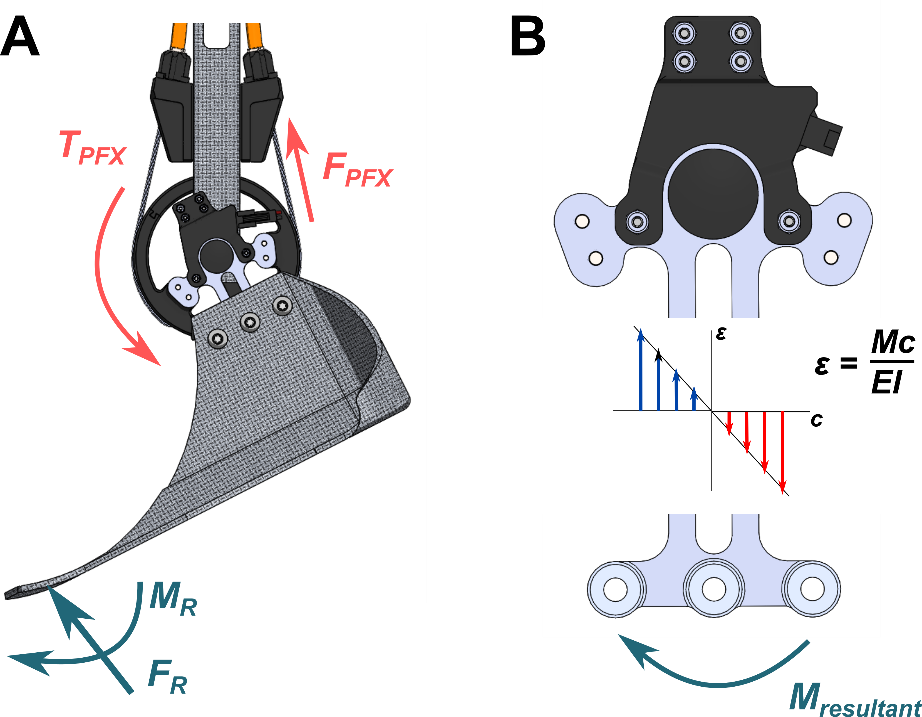 |
| --- |
| **Fig. S1.** Torque transducer function. **A.** The transducer transferred and measured torque between the pulley and footplate. **B.** The strain gauge configuration on the transducer measured the resultant sagittal bending moment between the footplate and pulley and negated out-of-plane bending and twisting. |

We used a calibrated load cell (LCM200, Futek) attached to a known lever arm to relate the applied torque to the transducer’s voltage response (Fig. S2, Additional File 1). Four transducers were loaded to 30 Nm three times in each direction. Five-point moving average data were collected at 100 Hz and offset by a zero-load calibration value taken at the beginning of each loading repetition in MATLAB (R2018b, MathWorks). Loading repetitions for each sensor were averaged. Data from three transducers were used as training data for a linear model estimating applied torque given a voltage reading. Data from the last transducer were used as a testing set to validate the model’s performance. The results of this validation were presented in the manuscript (Fig. 4A).

| 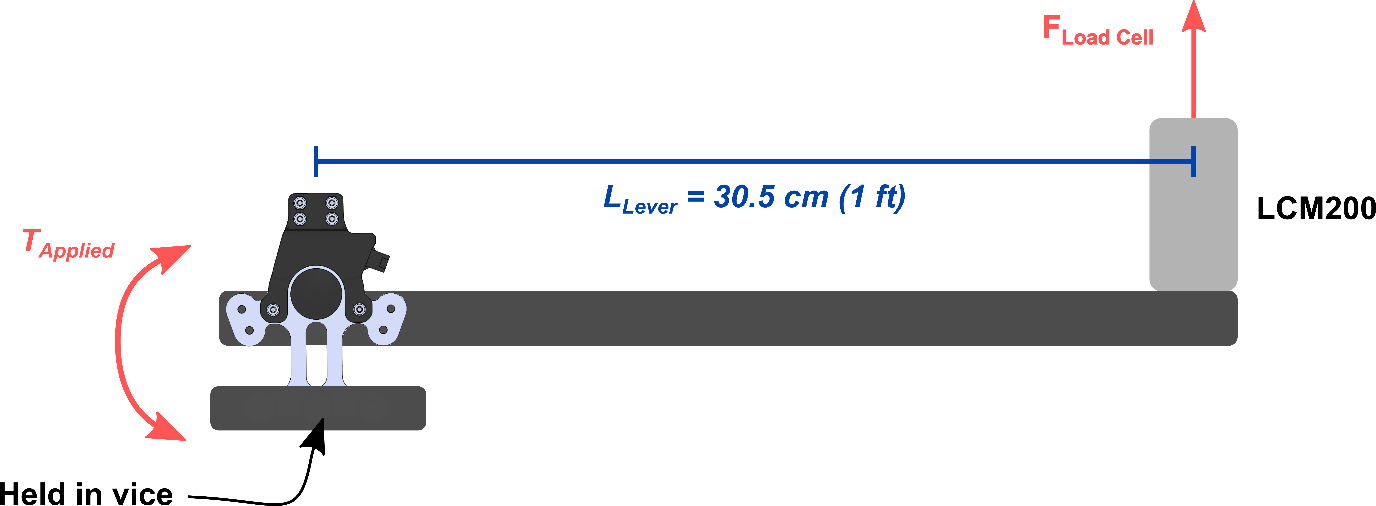 |
| --- |
| **Fig. S2.** Torque transducer validation setup. The LCM200 load cell was calibrated by the manufacturer and measured force. The torque transducer voltage response was related to the load cell force applied at the end of a lever using linear regression. Each transducer was loaded up to 30 Nm and unloaded three times in each direction. |

We also assessed the ability of the transducer to isolate sagittal plane moments (Additional File 1). Strain coupling with bending modes other than the primary load direction (i.e., sagittal bending in our case) can alter the sensor output voltage. To ensure that our torque sensor’s sensitivity to out-of-plane bending moments compared to the sagittal moment was low, we conducted an experiment to capture the sensor’s voltage response to each bending direction individually. One sensor was precisely loaded to 10 Nm and released three times in each plane to simulate the application of a bending moment (sagittal and coronal bending and axial twist) using the LCM200 load cell and calibration setup described in the previous section of this file. The torque sensor voltage response to each bending moment was determined using simple linear regression (Table S1, Figure S3). The torque sensor’s sensitivity to axial twist and coronal bending was 15.5% and 5.2% of the sagittal bending sensitivity, respectively.

| **Table S1. Torque sensor sensitivity regression fit summary.** The torque sensor sensitivity to coronal bending and axial twist is considerably less than for sagittal bending. Some error in the sensor voltage responses was likely due to the difficulty of perfectly isolating each bending mode. | | | | |
| --- | --- | --- | --- | --- |
| **Bending Mode** | **Sensor Sensitivity (V/Nm)** | **R^2^** | **Root-Mean-Squared Error (V)** | **Percent of Sagittal Sensitivity (%)** |
| Sagittal | 0.0233 | 0.998 | 0.0122 | - |
| Coronal | 0.0012 | 0.792 | 0.0022 | 5.2 |
| Axial Twist | 0.0036 | 0.930 | 0.0047 | 15.5 |

| 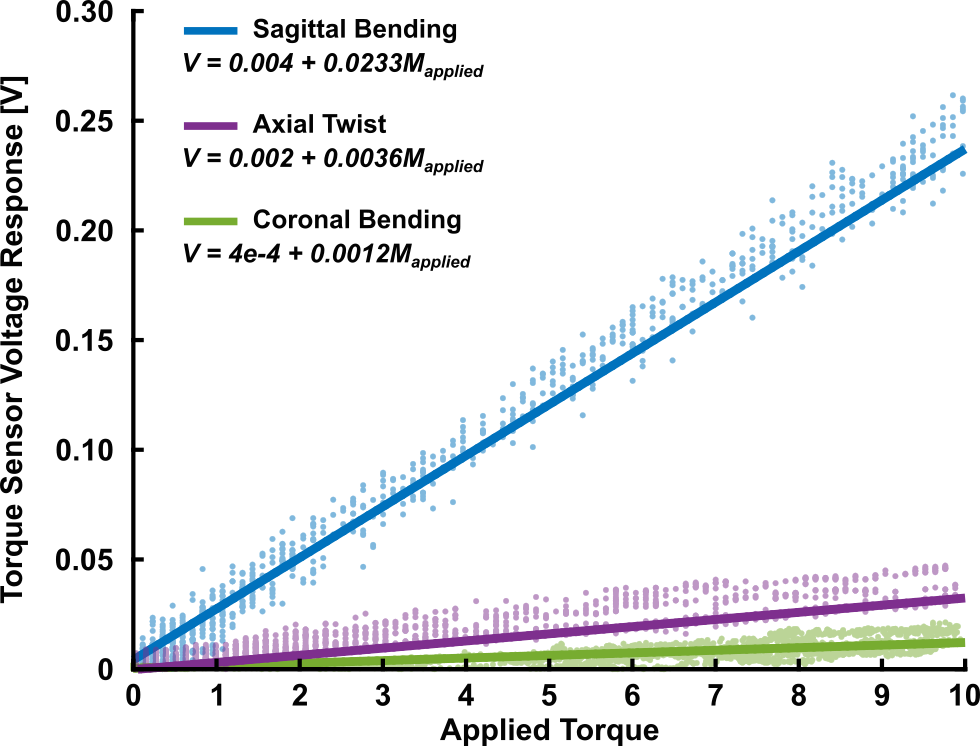 |
| --- |
| **Fig. S3. Comparison of torque sensor sensitivity to different bending modes.** The torque sensor was most sensitive to sagittal bending followed by axial twist and coronal bending. |

Angle Sensor Validation

We conducted a motion capture experiment to validate on-board angle and angular velocity measurement. 3D positions of retroreflective markers placed on bilateral ankle assemblies were recorded at 120 Hz in Vicon Nexus as the joints rotated in response to sinusoidal angular displacement driven by the motors (Fig. S5A). Motion capture data were filtered with a 4th order Butterworth low-pass filter with a 12 Hz cutoff, down-sampled to 100 Hz, and synchronized with a hardware trigger to the voltage data from each Hall sensor streamed over Bluetooth to MATLAB at 100 Hz. To validate the angular position measurement, 2/3 of the sensor voltage and corresponding motion capture angle data were randomly selected to generate a linear model. The training data spanned 40 degrees of exoskeleton joint motion split between plantarflexion and dorsiflexion regions. The remaining data were used to test the model and calculate the error between the sensor module measurement and the angles from motion capture. To validate on-board angular velocity estimation, the time derivative of the Hall sensor and motion capture angles were calculated by numerical differentiation. For comparisons and to confirm suspicions of mechanical delay due to the cable transmission, motor velocity was measured using the motor’s internal Hall sensors.

| 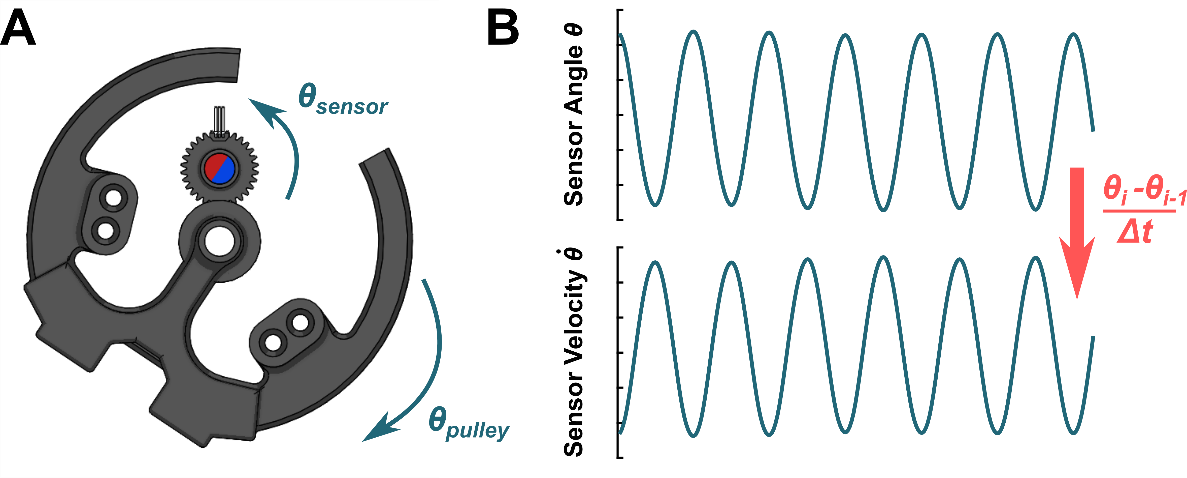 |
| --- |
| **Fig. S4.** Angle sensor function. **A.** Sagittal plane view of the angle sensor and pulley. The sensor shaft rotated opposite the pulley. A Hall sensor was held above the rotating diametric magnet. **B.** The Hall sensor output a repeatable voltage in response when the pulley was rotated. The pulley velocity was calculated in real time using a numerical derivative. |

| 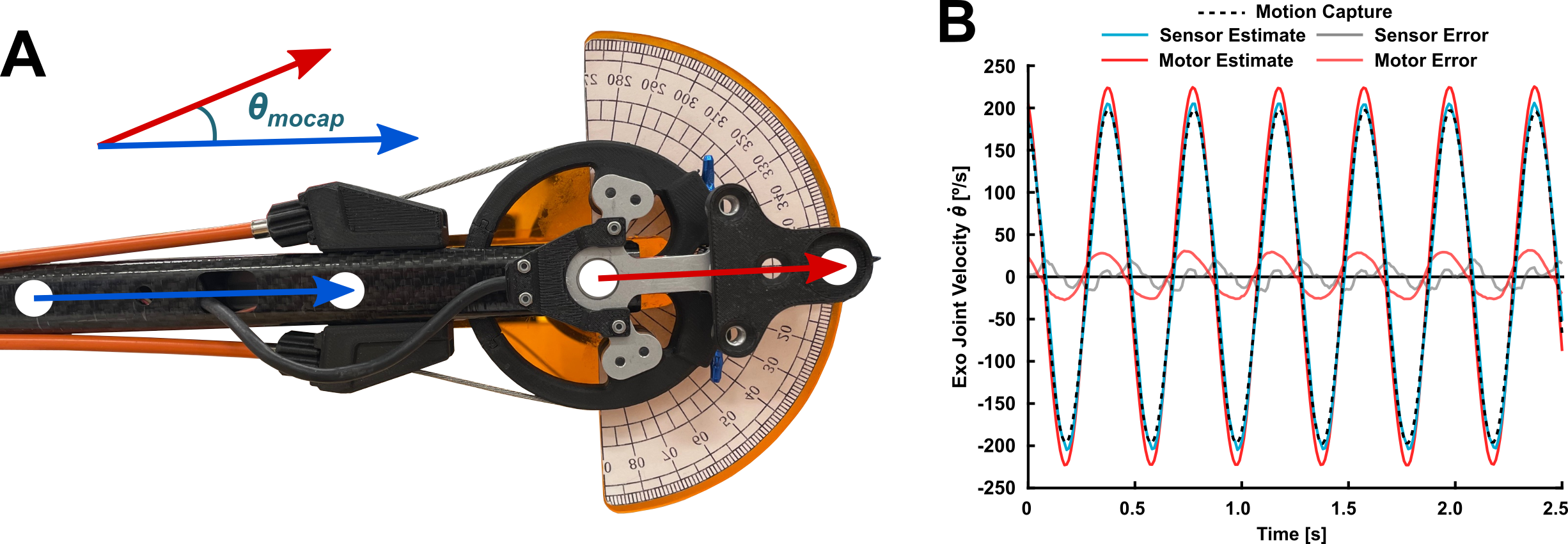 |
| --- |
| **Fig. S5.** Angle sensor validation setup. **A.** Reflective markers on the upright and exoskeleton joint defined two vectors in space such that angle between the vectors represented the angular position of the joint. The numerical derivative of the angle represented the angular velocity of the joint. **B.** A sample of angular velocity data from the validation experiment (same as Fig. 4 in the main text). The motors were commanded to move in a sinusoidal motion with velocity control while the custom angle sensor captured pulley angle and velocity. Some transmission losses between the motor and the pulley are evident as the motor estimate error is greater than the sensor estimate error (motor velocity RMSE = 17.85 deg/s, sensor velocity RMSE = 9.01 deg/s). |
