# Supplementary material for: Usability and performance validation of an ultra-lightweight and versatile untethered robotic ankle exoskeleton
Source: J Neuroeng Rehabil. 2021 Nov 10;18:163. doi: 10.1186/s12984-021-00954-9 (PMC8579560; doi:10.1186/s12984-021-00954-9)
Supplement: Supplementary file 2 — Additional file 2: Supplemental exoskeleton performance data. P4's torque, velocity, and power curves for inclined walking and stair ascent. Comparison of our device to other prominent exoskeleton studies. [file 12984_2021_954_MOESM2_ESM.docx]

| 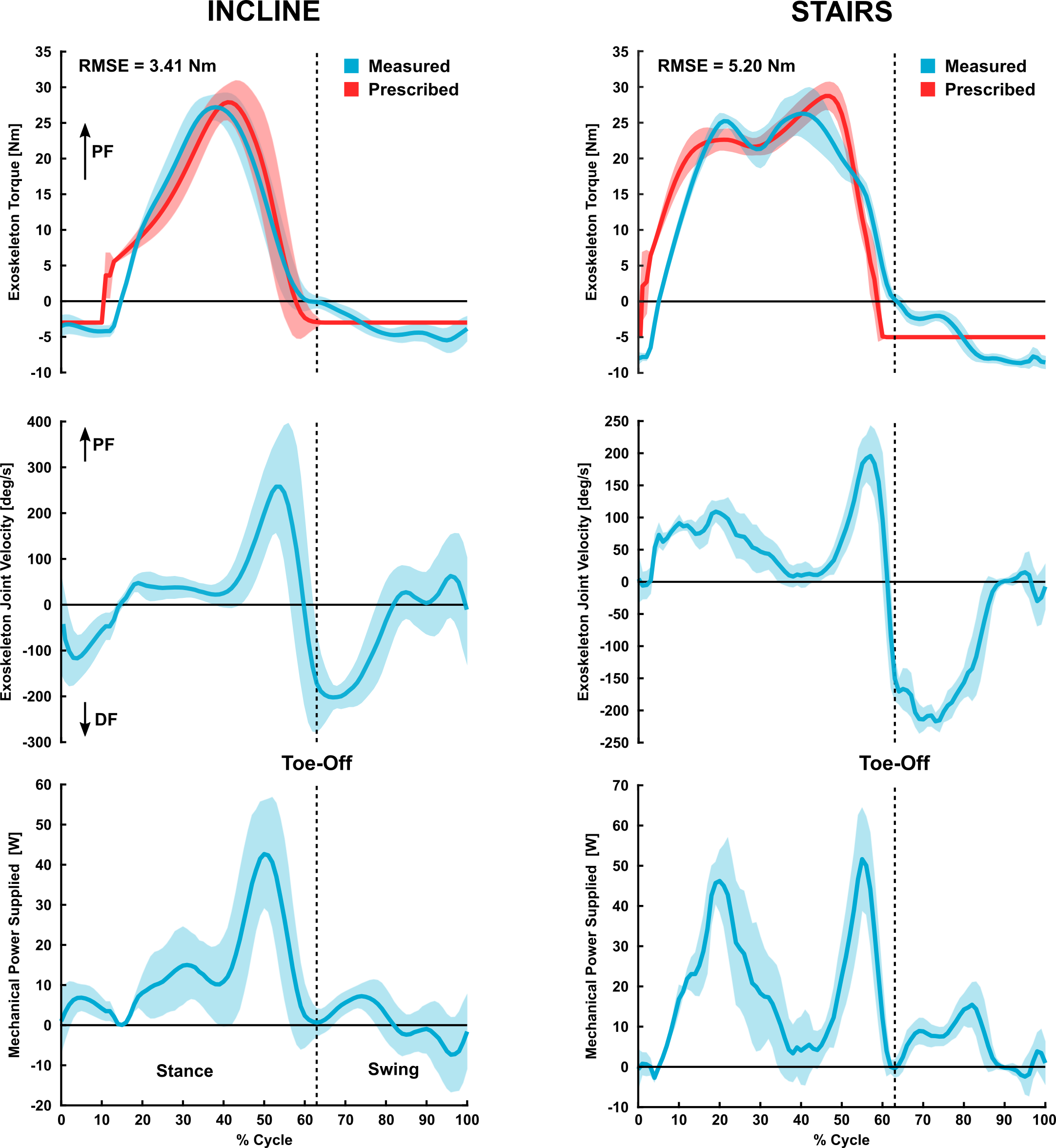 |
| --- |
| **Fig. S1.** Supplemental exoskeleton performance data. P4’s exoskeleton joint torque (top row), angular velocity (middle row), and mechanical power (bottom row) for inclined walking (left column) and stair ascent (right column). Peak measured torque typically ranged from 25 to 30 Nm. The upper torque capacity of the exoskeleton is 30 Nm. Torque root-mean-squared error (RMSE) was largely due to motor power limitations. The adaptive nature of our high-level controller results in variable torque, velocity, and power profiles between users and terrain. |

| 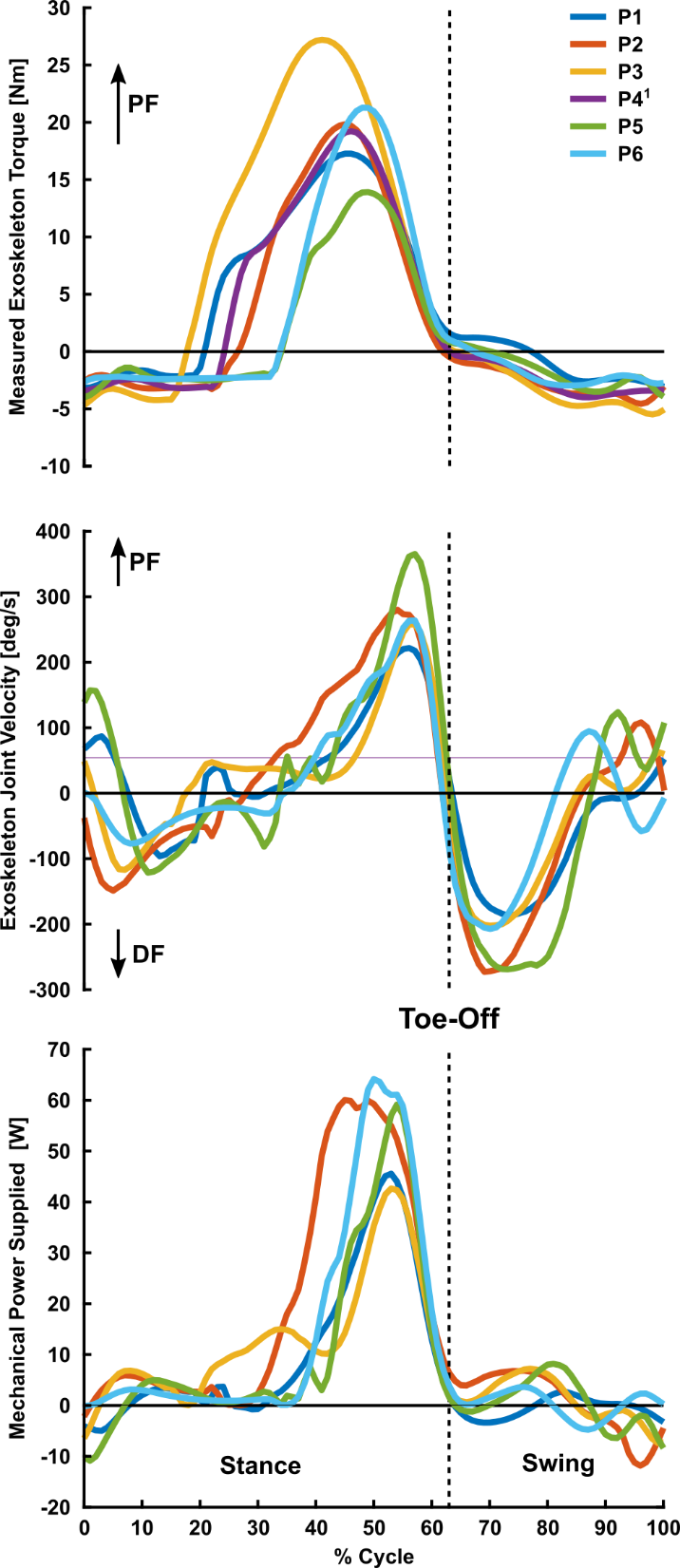 |
| --- |
| **Fig. S2.** Mean **e**xoskeleton measured joint torque (top), angular velocity (middle), and mechanical power (bottom) for each unimpaired participant. ^1^P4 did not have available angular velocity or joint power data. |

| 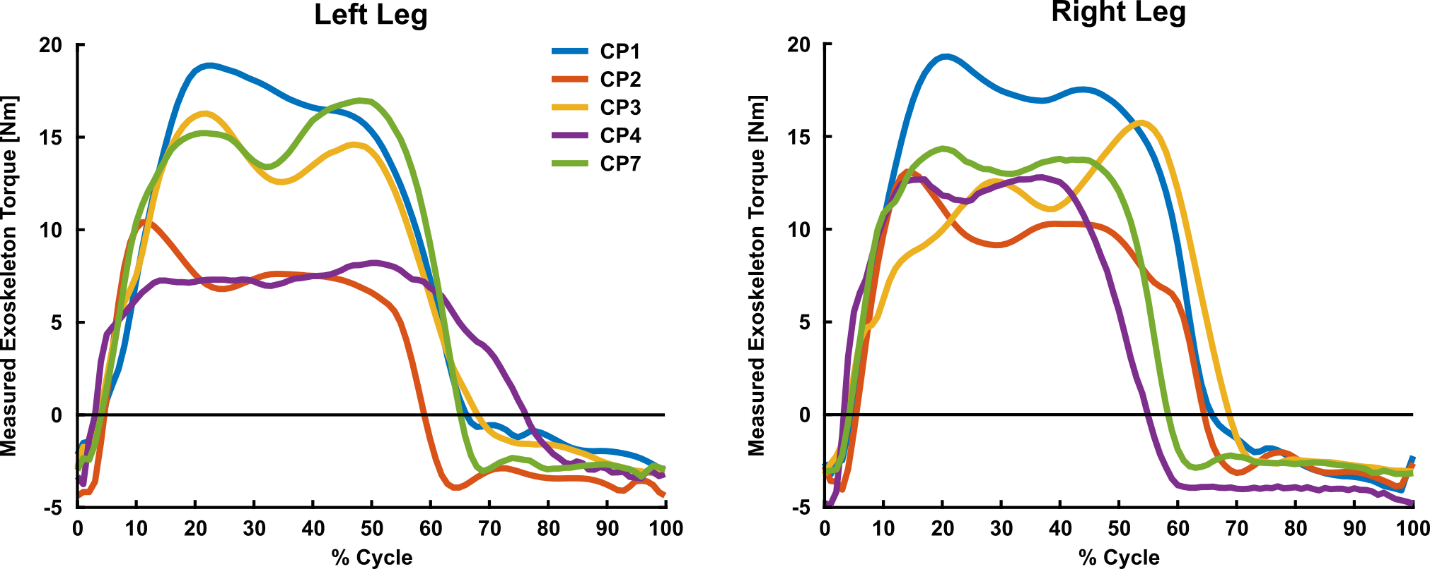 |
| --- |
| **Fig. S3.** Mean exoskeleton measured joint torque for the participants with CP that performed the stairs maximum exertion experiment. Joint angular velocity and mechanical power data were unavailable. |

| 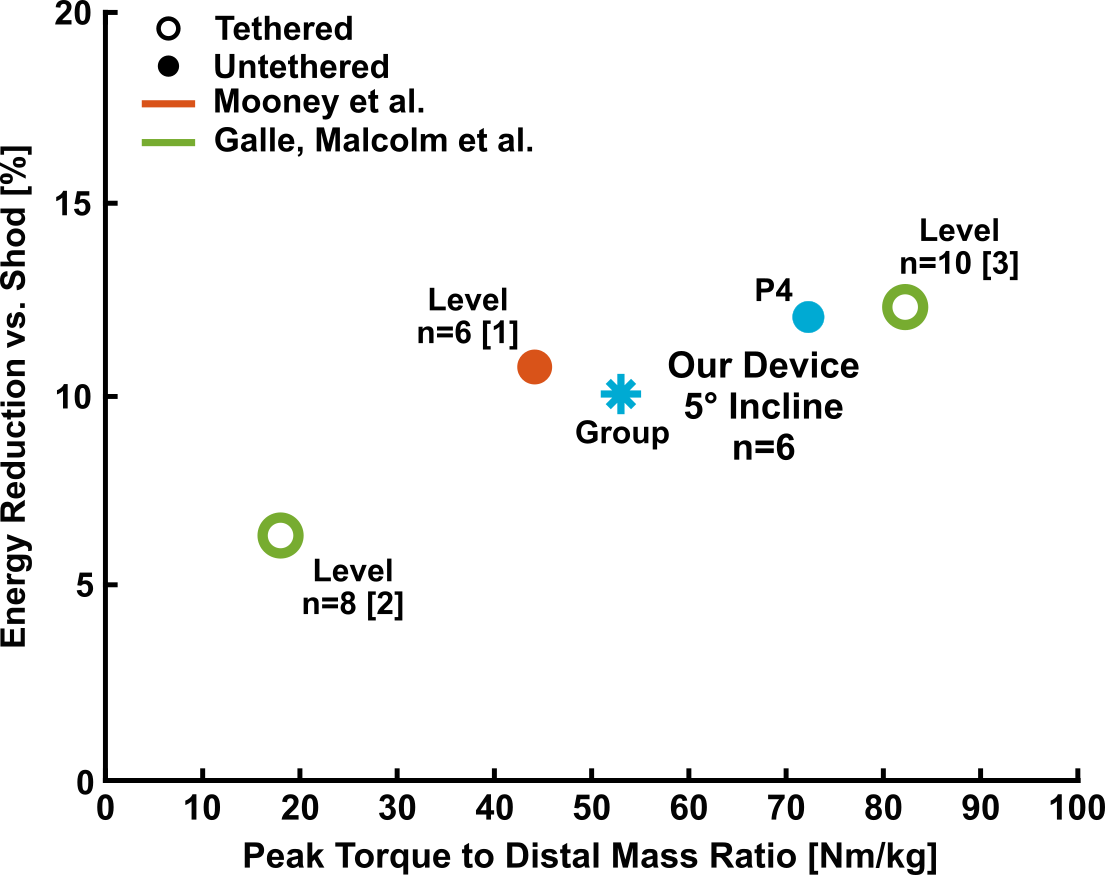 |
| --- |
| **Fig. S4.** Comparison of observed metabolic benefit for unimpaired users vs. peak exoskeleton torque to distal mass ratio. Data points with the same color represent the same exoskeleton design. Though our device had lower peak torque and power than some other devices, the low distal mass of our exoskeleton resulted in a high torque-to-weight ratio. The observed group-level reductions in metabolic power during assisted walking compared to no device was similar to other groups. Published studies demonstrating metabolic benefits during exoskeleton-assisted walking for unimpaired users are limited. |

References

1. Mooney LM, Herr HM. Biomechanical walking mechanisms underlying the metabolic reduction caused by an autonomous exoskeleton. J Neuroeng Rehabil. 2016;13(1).
2. Malcolm P, Derave W, Galle S, De Clercq D. A Simple Exoskeleton That Assists Plantarflexion Can Reduce the Metabolic Cost of Human Walking. PLoS One. 2013;8(2).
3. Galle S, Malcolm P, Collins SH, De Clercq D. Reducing the metabolic cost of walking with an ankle exoskeleton: interaction between actuation timing and power. J Neuroeng Rehabil. 2017;14(1).
