# Supplementary material for: Usability and performance validation of an ultra-lightweight and versatile untethered robotic ankle exoskeleton
Source: J Neuroeng Rehabil. 2021 Nov 10;18:163. doi: 10.1186/s12984-021-00954-9 (PMC8579560; doi:10.1186/s12984-021-00954-9)
Supplement: Supplementary file 3 — Additional file 3: Exoskeleton power consumption and electrcal-to-mechanical power efficiency analysis. [file 12984_2021_954_MOESM3_ESM.docx]

Power Consumption and Efficiency

The batteries used in this study had a capacity of 2000 mAh. To determine the effective battery life, overall power consumption, and electrical-to-mechanical efficiency of our device, we conducted an experiment in which one person walked on a treadmill inclined 5 degrees at 1.5 m/s with the exoskeleton unilaterally providing 22 Nm peak plantarflexor torque and 2.75 Nm dorsiflexor torque (the mean torque levels for the present study). Battery voltage, motor current, motor velocity, exoskeleton joint torque, and exo joint velocity were collected for 50 steps. To assess overall device power consumption, the motor current was integrated with respect to time for each step (Figure S1A). Exoskeleton joint mechanical power was calculated as the product of joint torque and angular velocity measured using our validated in-line sensors. Electrical power consumed by the device was calculated as the product of battery voltage measured by a voltage monitor (INA219, Texas Instruments) on the PCB and motor current measured by Maxon motor drivers. Electrical-to-mechanical power efficiency was calculated as the ratio of mean exoskeleton joint power output to mean electrical power consumed (Fig. S1B).

| 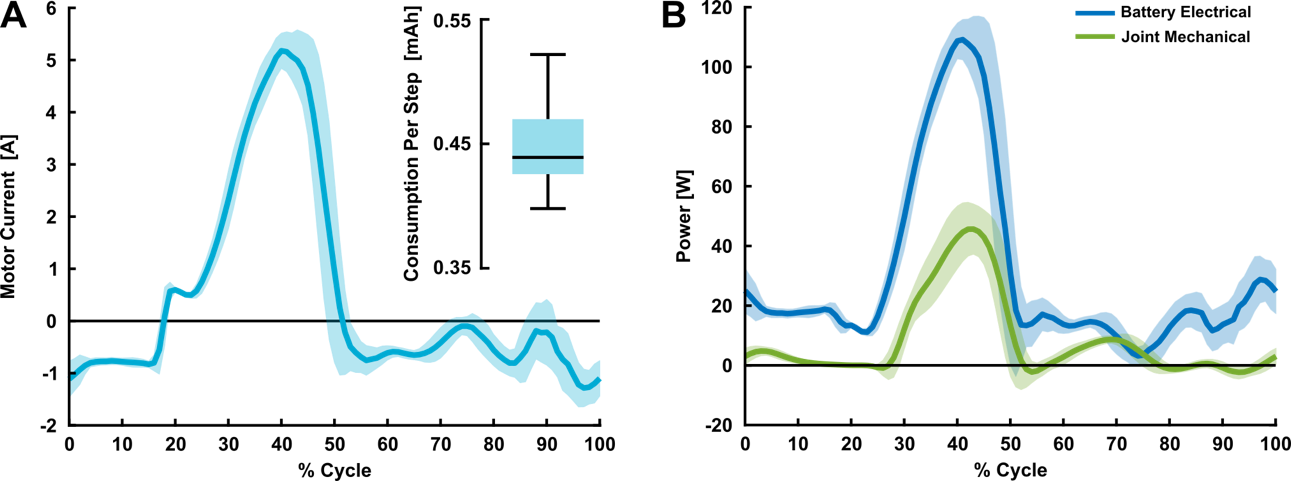 |
| --- |
| **Fig. S1. A.** Mean ± standard deviation motor current profile for one leg. In the corner, the distribution of power consumption per step in mAh is shown. The average power consumption per step was 0.45 ± 0.03 mAh which translates to about 2222 strides or 37 minutes if all 2000 mAh of the battery were used assuming a cadence of one stride per second. **B.** Electrical and mechanical power profiles for the battery and exoskeleton joint, respectfully. The electrical-to-mechanical power efficiency, calculated as the ratio of mean joint mechanical power to electrical power consumed during stance, was 35%. |
