# Supplementary material for: Usability and performance validation of an ultra-lightweight and versatile untethered robotic ankle exoskeleton
Source: J Neuroeng Rehabil. 2021 Nov 10;18:163. doi: 10.1186/s12984-021-00954-9 (PMC8579560; doi:10.1186/s12984-021-00954-9)
Supplement: Supplementary file 4 — Additional file 4: Video and instruction links. Links to videos of exoskeleton maximal exertion and usability experiments. Link to exoskeleton donning instructions. [file 12984_2021_954_MOESM4_ESM.docx]

**Supplemental Video 1.** CP2 maximal exertion experiment. DOI: https://doi.org/10.6084/m9.figshare.14810574

**Supplemental Video 2.** CP2 usability assessment. DOI: https://doi.org/10.6084/m9.figshare.14810598

**Supplemental Link 1.** Exoskeleton donning instructions on our website. Intended for viewing on iPhone. <https://biomech.nau.edu/don/>
