# Supplementary material for: Usability and performance validation of an ultra-lightweight and versatile untethered robotic ankle exoskeleton
Source: J Neuroeng Rehabil. 2021 Nov 10;18:163. doi: 10.1186/s12984-021-00954-9 (PMC8579560; doi:10.1186/s12984-021-00954-9)
Supplement: Supplementary file 5 — Additional file 5: Augmentation factor calculation for unimpaired cohort. [file 12984_2021_954_MOESM5_ESM.docx]

Augmentation Factor Calculation

Augmentation factor (AF) is an estimate of the potential metabolic impact an exoskeleton may have on a user [1,2]. As described in [1], the AF was developed for an autonomous ankle exoskeleton and estimates a change in metabolic power depending on device characteristics such as positive mechanical power, dissipated and mass distribution on the body. AF, defined as

|  | $AF= \frac{p^{+}+p^{dis}}{\eta}-\sum_{i=1}^{4} \beta_{i}m_{i}$ | (1) |
| --- | --- | --- |

where $p^{+}$was the mean positive power across the gait cycle, $p^{dis}$ was zero if the positive power exceeded the negative power or otherwise was the absolute value of the difference between positive and negative power, $\eta$ was the muscle-tendon efficiency, and $\beta_{i}$ was the metabolic impact of wearing added mass $m_{i}$ on body location *i* (Table S1). In short, AF balances the impact the exoskeleton mechanical power contribution ($p^{+}+p^{dis}$) has on a body based on muscle-tendon efficiency ($\eta$=0.41, [1,3,4]) with the overall detriment of wearing additional mass on the body ($\sum_{i=1}^{4} \beta_{i}m_{i})$. The metabolic detriment of wearing additional mass ($m_{i}$) varies by location on the body ($\beta_{i})$ with mass placed distally such as on the shank or foot having a greater impact on energetics than mass placed proximally on the pelvis (Table S1, [5]).

| **Table S1.** Exoskeleton component mass breakdown. Reprinted from the main text. | | | |
| --- | --- | --- | --- |
| **Component^1^** | **Mass (kg)** | **Location on Body** | **Metabolic Impact (W/kg)** |
| Waist assembly | 1.37 | Waist | 3.33 |
| Cable transmission (x2) | 0.31 | Thigh | 5.55 |
| Ankle assembly and cuff (x2) | 0.55 | Shank | 5.62 |
| Footplate (x2) | 0.28 | Foot | 14.80 |
| **Total Bilateral Exoskeleton Mass^3^** | 2.51 |  |  |
| ^1^ The mass corresponding to components indicated with (x2) is bilateral. ^2^ The impact of added mass on metabolic power based on location was investigated in Browning et. al. 2007 [5]. ^3^ The exoskeleton mass breakdown presented was for a medium-sized exoskeleton sized for users between 160 and 185 cm tall. | | | |

All participants in the unimpaired cohort used the same exoskeleton with small differences in the mass of cuffs and footplates. For simplicity, the same mass distribution as in Table S1 was used in calculating AF for each unimpaired participant. The metabolic detriment of wearing our exoskeleton was estimated to be 13.5 W. A positive AF predicts a reduction in energy expenditure when wearing an exoskeleton. Mean exoskeleton positive power $p^{+}$ was calculated by averaging the positive mechanical power data collected during the unimpaired cohort experiment for each stride across both legs (including both stance and swing phases) and averaging again across strides for each participant. As described in [1], if the exoskeleton mean positive power exceeds the mean negative power, $p^{dis}$ is zero. Refer to the exoskeleton joint power curves for each participant in Additional File 3 to observe that the positive mechanical power for each stride greatly exceeds any negative mechanical power. Components of the AF calculation in Eq. 1 are summarized in Table S2 for each participant. Each participant’s actual observed metabolic impact is also summarized.

| **Table S2.** Summary of unimpaired participant characteristics, AF calculation components, and the predicted and actual metabolic benefit from the experiment in the main text. | | | | | | | |
| --- | --- | --- | --- | --- | --- | --- | --- |
| **Participant** | **Mass (kg)** | **Peak Stance Torque (Nm)** | **p^+^ (W)^1^** | **p^dis^ (W)** | **Mass Detriment (W)** | **Augmentation Factor (W)** | **Metabolic Impact (W)** |
| P1 | 50.0 | 17.5 | 17.1 | 0 | 13.5 | 28.1 | 38.0 |
| P2 | 57.5 | 20.5 | 28.3 | 0 | 13.5 | 55.6 | 35.1 |
| P3 | 91.6 | 30.0 | 20.8 | 0 | 13.5 | 37.2 | 81.5 |
| P4^2^ | 65.0 | 23.0 | - | - | - | - | 78.3 |
| P5 | 45.7 | 16.0 | 21.3 | 0 | 13.5 | 38.4 | 0.1 |
| P6 | 72.6 | 25.0 | 20.1 | 0 | 13.5 | 35.4 | 90.9 |
| ^1^The mean positive power, dissipated power, mass detriment, and augmentation factor are bilateral. ^2^ Mechanical power data was not collected for P4. | | | | | | | |

For all participants, dissipated power $p^{dis}$ was zero and the AF was positive, meaning that all participants should use less energy when walking with the exoskeleton. While all but one participant had a marked improvement in metabolic power when walking with exoskeleton assistance compared to shod, the agreement between the benefit predicted by AF and the actual observed benefit was poor References

1. Mooney LM, Rouse EJ, Herr HM. Autonomous exoskeleton reduces metabolic cost of human walking during load carriage. J Neuroeng Rehabil. 2014; 11(80).

2. Mooney LM, Rouse EJ, Herr HM. Autonomous exoskeleton reduces metabolic cost of human walking. J Neuroeng Rehabil. 2014; 11(151).

3. Sawicki GS, Ferris DP. Powered ankle exoskeletons reveal the metabolic cost of plantar flexor mechanical work during walking with longer steps at constant step frequency. J Exp Biol. 2009; 212(1): 21–31.

4. Malcolm P, Derave W, Galle S, De Clercq D. A Simple Exoskeleton That Assists Plantarflexion Can Reduce the Metabolic Cost of Human Walking. PLoS One. 2013; 8(2).

5. Browning RC, Modica JR, Kram R, Goswami A. The effects of adding mass to the legs on the energetics and biomechanics of walking. Med Sci Sports Exerc. 2007; 39(3): 515–25.
